# DNA barcoding and phylogenetic analysis to characterize biodiversity of some freshwater fish species in Lake Nasser and River Nile

**DOI:** 10.1038/s41598-025-20830-z

**Published:** 2025-10-09

**Authors:** Esraa Mostafa Ashour, Ragaa A. Ahmed, Nermeen Y. Abass

**Affiliations:** 1 Department of Agricultural Botany, Faculty of Agriculture Saba-Basha, Alexandria University, P.O. Box 21531, Alexandria City, Egypt; 2 Department of Aquaculture, Faculty of Fish and Fisheries Technology, Aswan University, Aswan, Egypt

**Keywords:** DNA barcoding, Phylogenetic, Freshwater fish, Lake Nasser, River Nile, Biodiversity, Ecology, Ecology, Evolution, Genetics, Molecular biology, Zoology

## Abstract

The present study aims to provide an inventory of eight freshwater fish species that represent 6 families obtained from Lake Nasser and the River Nile using DNA barcoding, using analysis of cytochrome oxidase subunit I (COI) gene sequences. Fish samples were identified morphologically as *Ctenopharyngodon idella*, *Oreochromis niloticus*, *Bagrus bajad*, *Sarotherodon galilaeus*, *Auchenoglanis occidentalis*, *Lates niloticus*, *Sardinella tawilis*, and *Coptodon zillii*. COI gene was successfully amplified for using PCR, showing a readable fragment length of ~ 700 base pairs (bp). The obtained sequences were compared to GenBank and the Barcode of Life Data System (BOLD). The GenBank and BOLD resulted in a total of 6 fish samples finding their best hits with similarity scores ranging from 99.29 to 100%. The average AT content (53.12%) was higher than the average GC content (46.88%) in the studied fish species. The minimum genetic Kimura 2-parameter (K2P) distance between species was 0.089 and the maximum distance was 0.313. According to phylogenetic tree analysis, the majority of fish species were grouped into monophyletic units. The current study confirms that DNA barcoding is a useful method to identify the vast majority of fish species in Lake Nasser and the River Nile. However, the morphological identification remains crucial.

**Supplementary Information:**

The online version contains supplementary material available at 10.1038/s41598-025-20830-z.

## Introduction

In Africa and worldwide, the River Nile is the longest river. It flows as a vast irrigation system with numerous interconnected canals, creating the most well-known urban life in the Nile valley and Delta’s history. The River Nile is essential to Egypt’s hydroelectric power, drinking water, irrigation, and fish farming^1^. However, the River Nile and its numerous canals provide very little freshwater fisheries productivity^2^. The River Nile and its branches are considered essential freshwater fish species by millions of Egyptians who reside in the little villages scattered on the Nile sides^3,4^.

In Egypt, the River Nile has been shown to have high levels of pollution^5^, besides, overexploitation of its fish resources which may have a significant impact on freshwater fish species^6^. According to reports, the most endangered group of vertebrates is freshwater fish^7^. The populations of certain species declined and getting to be extinct at a fast rate^8^. In addition, a lot of fish species may become extinct before they can be recorded^8^. The extinction of certain species will significantly impact the management of the genetic resources since these species could not be protected effectively^9^.

In Africa, the Aswan Reservoir is one of the largest artificial freshwater lakes in the world. The reservoir is approximately 496 km in length and nearly 35 km in width. The Lake Nasser is roughly 292 km long in Egypt. The aquatic animals’ community of Lake Nasser contains 52 species from 15 families of fishes^10^. Lake Nasser’s capture fisheries accounted for over 66% of Egypt’s entire output from inland lakes^11^. Lake Nasser is an important source of freshwater fish species in Upper Egypt^12^.

The number of freshwater fish species living along Egypt’s River Nile dropped from 85 in 1907 to 71 in 1997^13^. In 1964, a total of 57 species from 16 families were identified in Lake Nasser and the River Nile^14^. After over four decades, the diversity of freshwater fish species declined and some fish species became limited to certain areas of Lake Nasser, while others have disappeared completely^14^. Totally 29 freshwater fish species belonging to 13 families were recorded along the Egyptian Nile in 2010 reaching only 35 freshwater fish species belonging to 13 fish families nowadays^13^. Due to this noted decline of the freshwater fish biodiversity in Egypt, it becomes essential to monitor these species inventory, genetic diversity, and population structure.

Fishes are vital aquatic animals of great diversity in morphological appearances and they include several broad classes collectively representing the greatest diversity of vertebrates (> 35,000 species)^15,16^. The variety of sizes, colours, forms, and habitats is astounding. Fish diversity is greater than that of any other group of vertebrates^17^.

Fish identification is not only of interest for systematists and taxonomy, but it is essential for maintaining biodiversity, food and drug safety, natural history and ecology, and sustainable fishery management^17,18^. Numerous approaches have been used to identify fish species, including conventional morphological identification, environmental DNA analysis, next-generation sequencing (NGS), nanopore sequencing, and DNA barcoding^19^.

Due to high diversity and morphological plasticity, in many cases, fish and their different developmental stages are often difficult to identify by using morphological characteristics alone^20^. Identification methods based on DNA have been developed and shown to be highly analytically effective. DNA-barcoding technology is the best molecular method for identifying fish species^21^. Numerous investigations have effectively identified fish species using DNA barcoding, demonstrating its accessibility, speed, and dependability^21–23^. DNA barcoding has been shown to be beneficial for managing fisheries properly and for elucidating the cryptic lineages found in many fish species^21,24^.

Many studies have indicated that cytochrome oxidase subunit I (COI) barcode marker accurately differentiate between closely related species compared to other DNA barcodes^21,25^. A 650 bp fragment at the 5’ end of COI gene is used to differentiate most animal species in general^25,26^ and fish species in particular^21^. The fragment at the COI gene 5’ end is considered an effective marker for species classification as it has higher sequence divergence between species compared to within species divergence^26^.

Different molecular identification techniques, such as neighbor-joining (NJ) trees, Barcode Index Number, and Automatic Barcode Gap Discovery (ABGD), were used by Ali et al.^21^ to identify fish species in Egypt. In addition to morphological identification, 160 DNA barcodes were generated from 37 species, representing 32 genera, 15 families, and 9 orders, using a 655-bp-long fragment of the mitochondrial COI gene. Different families had different nucleotide diversity (π), with the families Malapteruridae, Auchenoglanididae, Schilbeidae, Anguillid, Centropomidae, and Tetraodontidae having 0.0% and the family Cyprinidae having 17%.

The pairwise genetic distance estimations of Nigerian freshwater fishes were 10.29% among congeners and just 0.17% among conspecifics, indicating that the average genetic distance was 60 times greater across species than within species. Despite low levels of divergence within species^27^.

The objectives of the current study are to assess the genetic diversity of freshwater fish species and provide an inventory of some fish species in Egypt using DNA barcoding. The results will be used to inform conservation efforts for River Nile fish species in Egypt and other Nile basin countries.

## Results

### Morphology identification

All the collected fish were identified based on morphology. The morphological identification classified fish into 8 species belonging to 6 families, and 5 orders (Table 1). The Fishbase database^28^ and the Barcode of Life Data System (BOLD), https://v3.boldsystems.org, were used to classify the studied species. The majority of fish species observed in our study belonged to the Cichlidae family (Table 1).

**Table 1:** Classification and sample size of the fish species used in this study.

No	Specific name	Genus	Family	Order	Common name	Sampling location	Sample size
1	*Ctenopharyngodon idella*	*Ctenopharyngo*	Cyprinidae	Cypriniformes	Grass carp	River Nile	3
2	*Oreochromis niloticus*	*Orechromis*	Cichlidae	Cichliformes	Nile tilapia		3
3	*Bagrus bajad*	*Bagrus*	Bagridae	Siluriformes	Bayad catfish		3
4	*Sarotherodon galilaeus*	*Sarotherodon*	Cichlidae	Cichliformes	Mango tilapia	Lake Nasser	3
5	*Auchenoglanis occidentalis*	*Auchenoglanis*	Claroteidae	Siluriformes	Yellow giraffe catfish		3
6	*Lates niloticus*	*Lates*	Latidae	Perciformes	Nile perch		3
7	*Bagrus bajad*	*Bagrus*	Bagridae	Siluriformes	Bayad catfish		3
8	*Sardinella tawilis*	*Sardinella*	Clupeidae	Clupeiformes	Bombon sardine		3
9	*Coptodon zillii*	*Coptodon*	Cichlidae	Cichliformes	Tilapia zillii		3
10	*Oreochromis niloticus*	*Orechromis*	Cichlidae	Cichliformes	Nile tilapia		3

### Identification of fish species using DNA barcodes

**Fig. 1:**
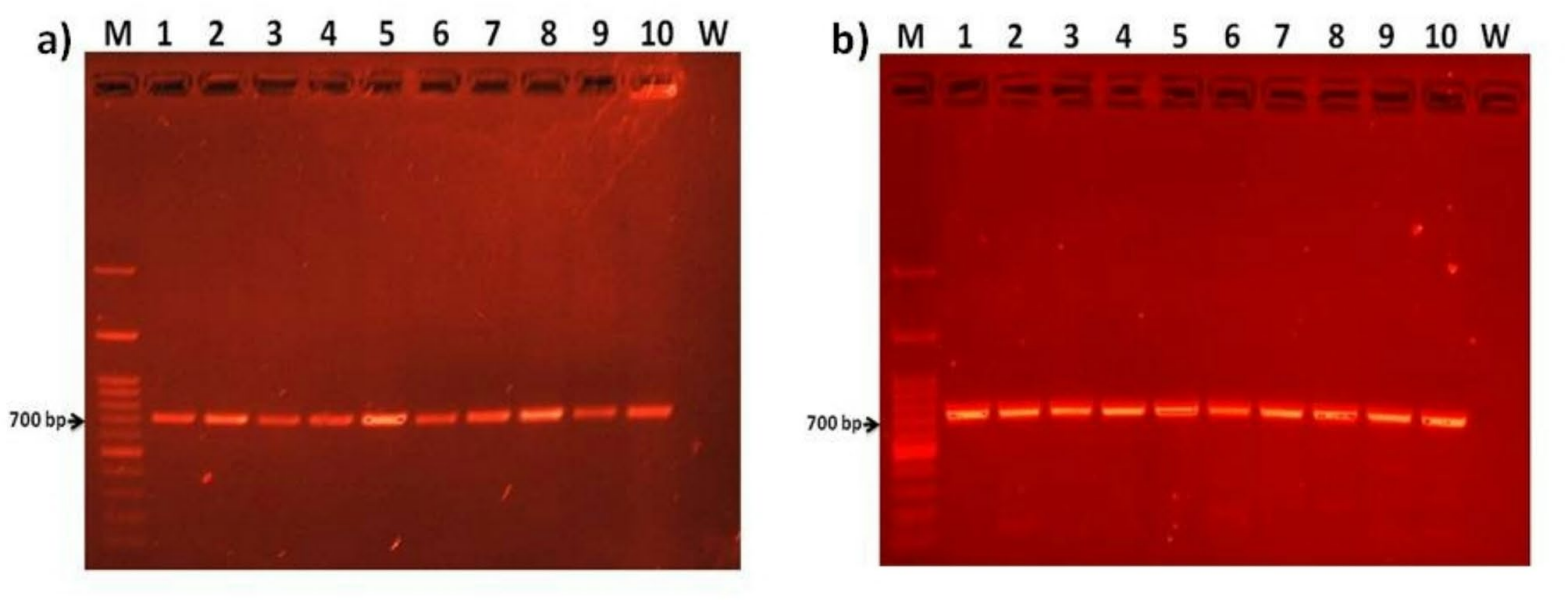
PCR analyses of the cytochrome oxidase subunit I (COI) gene of ten representative freshwater fish samples from Lake Nasser and the River Nile, total DNA. M = marker, marker lanes contain 1 Kb plus DNA ladders (Invitrogen); 1 =  *Ctenopharyngodon Idella*; 2 = *Oreochromis niloticus*; 3 = *Bagrus bajad*; 4 = *Sarotherodon galilaeus*; 5 = *Auchenoglanis occidentalis;* 6 = *Lates niloticus*; 7 = *Bagrus bajad*; 8 = *Sardinella tawilis*; 9 = *Coptodon zillii*; 10 = *Oreochromis niloticus*; and W = water control, PCR reaction of water served as negative control. 1–3 samples from the River Nile and 4–10 samples from Lake Nasser. (**a**) analysis by using primers COI Fish-F/COI Fish-R; and (**b**) analysis by using primers FishF1/FishR1.

The mitochondrial COI region of ten representative freshwater fish samples from Lake Nasser and the River Nile was successfully amplified using PCR which has product size of around 700 bp (Fig. 1). Table 2 shows the comprehensive barcoding identification results based on GenBank and the BOLD databases. The reference sequences of GenBank for the identification of all freshwater fish species were *Ctenopharyngodon idella* (OP680615 and OP680616), *Oreochromis niloticus* **(**OP680705 and OP680615 for the River Nile, and OP680615 and OP680616 for Lake Nasser), *Bagrus bajad* (MN509596 and MN509598 for the River Nile and LC487125 for Lake Nasser), *Sarotherodon galilaeus* (KM438546), *Auchenoglanis occidentalis* (HM882801), *Lates niloticus* (ON247427 and OL804282), *Sardinella tawilis* (HM882880), and *Coptodon zillii* (MG438464 and MG438465) (Table 2). The BIN number of BOLD for the identification of freshwater fish species were *Oreochromis niloticus* (GBAAX41364-24 and GBAAX41411-24 for the River Nile, and GBAAX41362-24 and GBAAX41362-25 for Lake Nasser), *Bagrus bajad* (UKFBJ231-08 and ANGBF43998-19 for the River Nile and GBAAX43494-24 for Lake Nasser), *Sarotherodon galilaeus* (FFMBH1029-14), *Auchenoglanis occidentalis* (ANGBF52972-19 and ANGBF14732-19), *Lates niloticus* (GBMNF49338-22), *Sardinella tawilis* (BAFEN203-10), and *Coptodon zillii* (ANGBF34374-19) (Table 2). Both databases revealed definitive identity matches in the range of 99.29%–100% for consensus sequences of five species (*Oreochromis niloticus*, *Sarotherodon galilaeus*, *Auchenoglanis occidentalis*, *Lates niloticus*, and *Coptodon zillii*). GenBank-based identification for all fish species yielded an alignment E-value of 0.0. *Oreochromis niloticus* from both locations, *Sarotherodon galilaeus, Auchenoglanis occidentalis*, *Lates niloticus,* and *Coptodon zillii* had 100% maximum identity in GenBank and BOLD database. GenBank and BOLD failed to discriminate *Ctenopharyngodon idella*, *Bagrus bajad*, and *Sardinella tawilis*. At the time of analysis, GenBank results were in agreement with BOLD results in identification of *Bagrus bajad*, and *Sardinella tawilis*. The GenBank and BOLD database hit using our *Sardinella tawilis* sequences was a single *Alestes baremoze* sequence (100% identity), *Bagrus bajad* sequences from Lake Nasser was a single *Chrysichthys auratus* (99.70–100% identity), and *Bagrus bajad* sequences from the River Nile was a single *Schilbe intermedius* (99.84–100% identity). No match was garnered for *Ctenopharyngodon idella* from BOLD, while GenBank, lacking a *Ctenopharyngodon idella* sequence, returned a top hit for a related species, *Oreochromis niloticus* (90.91% identity) (Table 2).

**Table 2:** Summary of fish identification based on each species consensus barcoded sequence using BLASTN search from GenBank and the Barcode of Life Data System (BOLD).

Species studied	No	Sampling location	GenBank (BLASTN)			BOLD		
			Species identification	% Max identity	Accession number	Species identification	% Max Similarity	BIN (Cluster ID)
*Ctenopharyngodon idella*	1	River Nile	Oreochromis niloticus*	90.91	OP680615	No match*	–	–
	2		Oreochromis niloticus*	92.47	OP680615	No match*	–	–
	3		Oreochromis niloticus*	90.91	OP680616	No match*	–	–
*Oreochromis niloticus*	1		*Oreochromis niloticus*	100	OP680705	*Oreochromis niloticus*	100	GBAAX41364-24
	2		*Oreochromis niloticus*	100	OP680615	*Oreochromis niloticus*	100	GBAAX41411-24
	3		*Oreochromis niloticus*	100	OP680705	*Oreochromis niloticus*	100	GBAAX41364-24
*Bagrus bajad*	1		Schilbe intermedius*	99.84	MN509596	Schilbe intermedius*	100	UKFBJ231-08
	2		Schilbe intermedius*	99.84	MN509596	Schilbe intermedius*	100	UKFBJ231-08
	3		Schilbe intermedius*	100	MN509598	Schilbe intermedius*	100	ANGBF43998-19
*Sarotherodon galilaeus*	1	Lake Nasser	*Sarotherodon galilaeus*	99.85	KM438546	*Sarotherodon galilaeus*	99.85	FFMBH1029-14
	2		*Sarotherodon galilaeus*	100	KM438546	*Sarotherodon galilaeus*	100	FFMBH1029-14
	3		*Sarotherodon galilaeus*	99.85	KM438546	*Sarotherodon galilaeus*	99.85	FFMBH1029-14
*Auchenoglanis occidentalis*	1		*Auchenoglanis occidentalis*	99.85	HM882801	*Auchenoglanis occidentalis*	100	ANGBF52972-19
	2		*Auchenoglanis occidentalis*	100	HM882801	*Auchenoglanis occidentalis*	100	ANGBF14732-19
	3		*Auchenoglanis occidentalis*	100	HM882801	*Auchenoglanis occidentalis*	100	ANGBF14732-19
*Lates niloticus*	1		*Lates niloticus*	100	ON247427	*Lates niloticus*	100	GBMNF49338-22
	2		*Lates niloticus*	100	ON247428	*Lates niloticus*	100	GBMNF49338-22
	3		*Lates niloticus*	100	OL804282	*Lates niloticus*	100	GBMNF49338-22
*Bagrus bajad*	1		Chrysichthys auratus*	99.70	LC487125	Chrysichthys auratus*	100	GBAAX43493-24
	2		Chrysichthys auratus*	99.70	LC487125	Chrysichthys auratus*	100	GBAAX43493-24
	3		Chrysichthys auratus*	99.54	LC487125	Chrysichthys auratus*	100	GBAAX43493-24
*Sardinella tawilis*	1		Alestes baremoze*	100	HM882880	Alestes baremoze*	100	BAFEN203-10
	2		Alestes baremoze*	100	HM882880	Alestes baremoze*	100	BAFEN203-10
	3		Alestes baremoze*	100	HM882880	Alestes baremoze*	100	BAFEN203-10
*Coptodon zillii*	1		*Coptodon zillii*	98.86	MG438464	*Coptodon zillii*	98.86	ANGBF34374-19
	2		*Coptodon zillii*	98.86	MG438465	*Coptodon zillii*	98.86	ANGBF34374-19
	3		*Coptodon zillii*	100	MG438465	*Coptodon zillii*	100	ANGBF34374-19
*Oreochromis niloticus*	1		*Oreochromis niloticus*	99.29	OP680615	*Oreochromis niloticus*	100	GBAAX41362-24
	2		*Oreochromis niloticus*	100	OP680616	*Oreochromis niloticus*	100	GBAAX41362-24
	3		*Oreochromis niloticus*	100	OP680616	*Oreochromis niloticus*	100	GBAAX41362-25

*Bolded words with asterisk correspond to problematic identifications of fish species in the current study using either one or both of the databases.

**Table 3:** DNA sequence analysis, mean base composition ± SD, and the average AT and GC nucleotide composition in the studied fish samples.

Species	Sampling location	A	T	G	C	Total (bp)	AT (%)	GC (%)
*Ctenopharyngodon idella*	River Nile	165.67 ± 2.89^bc^	220.33 ± 1.15^a^	120.00 ± 0.00^cd^	199.00 ± 1.73^b^	705.00 ± 0.00^a^	54.75 ± 0.25^b^	45.25 ± 0.25^e^
*Oreochromis niloticus*		175.00 ± 0.00^a^	199.67 ± 0.58^c^	120.33 ± 0.58^cd^	210.00 ± 0.00^a^	705.00 ± 0.00^a^	53.14 ± 0.08^d^	46.86 ± 0.08^c^
*Bagrus bajad*		168.33 ± 0.58^b^	170.00 ± 1.73^g^	111.00 ± 0.00^e^	195.33 ± 0.58^b^	644.67 ± 2.89^de^	52.48 ± 0.12^e^	47.52 ± 0.12^b^
*Sarotherodon galilaeus*	Lake Nasser	156.33 ± 0.58^d^	190.00 ± 0.00^d^	117.67 ± 0.58^ cd^	196.00 ± 0.00^b^	660.00 ± 0.00^bc^	52.47 ± 0.09^e^	47.53 ± 0.09^b^
*Auchenoglanis occidentalis*		163.33 ± 0.58^c^	183.33 ± 1.53^f^	117.00 ± 0.00^d^	188.67 ± 0.58^c^	652.33 ± 1.53^cd^	53.14 ± 0.07^d^	46.86 ± 0.07^c^
*Lates niloticus*		154.67 ± 4.62^d^	186.67 ± 4.62^e^	111.33 ± 5.77^e^	183.33 ± 5.77^d^	636.00 ± 20.78^e^	53.68 ± 0.30^c^	46.32 ± 0.30^d^
*Bagrus bajad*		163.67 ± 2.31^c^	191.67 ± 2.31^d^	130.33 ± 1.15^b^	182.00 ± 5.20^b^	667.67 ± 10.97^b^	53.22 ± 0.18^d^	46.78 ± 0.18^c^
*Sardinella tawilis*		153.00 ± 0.00^d^	209.00 ± 0.00^b^	118.00 ± 0.00^cd^	171.33 ± 0.58^e^	651.33 ± 0.58^cd^	55.58 ± 0.05^a^	44.42 ± 0.05^f^
*Coptodon zillii*		156.00 ± 0.00^d^	191.00 ± 1.73^d^	138.33 ± 2.89^a^	213.67 ± 4.04^a^	699.00 ± 8.66^a^	49.65 ± 0.37^f^	50.35 ± 0.37^a^
*Oreochromis niloticus*		174.67 ± 0.58^a^	199.00 ± 0.00^c^	121.33 ± 0.58^c^	209.00 ± 1.73^a^	704.00 ± 1.73^a^	53.08 ± 1.15^d^	46.92 ± 0.05^c^
Average							53.12 ± 1.51	46.88 ± 1.51

Within a column, means that do not differ at *P* = 0.05 are followed by the same superscript (Duncan’s multiple range test) among different tissues for each group.

### Phylogenetic analysis

**Fig. 2:**
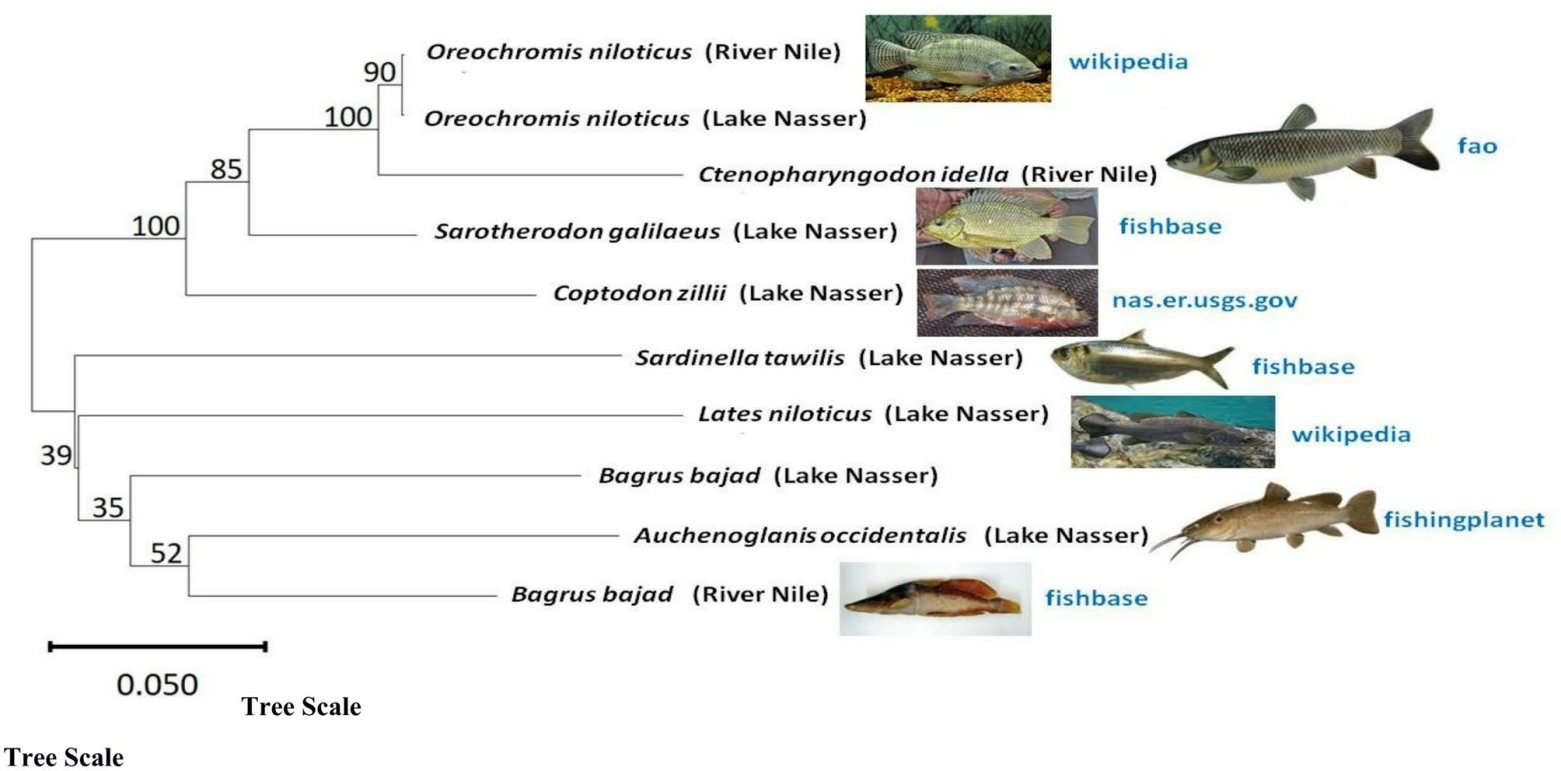
Phylogenetic tree of ten representative freshwater fish samples from Lake Nasser and the River Nile prepared using MEGA version 12.0. Phylogenetic analyses of the studied species were performed based on Neighbor-Joining (NJ) and maximum parsimony (MP) methods. The % of replicate tree in which the associated taxa clustered together in the bootstrap test (1000 replicates) is shown next to the branches. The source for each image was indicated next to the pictures. Scale bars represent 0.050 nucleotide substitutions per site.

**Fig. 3:**
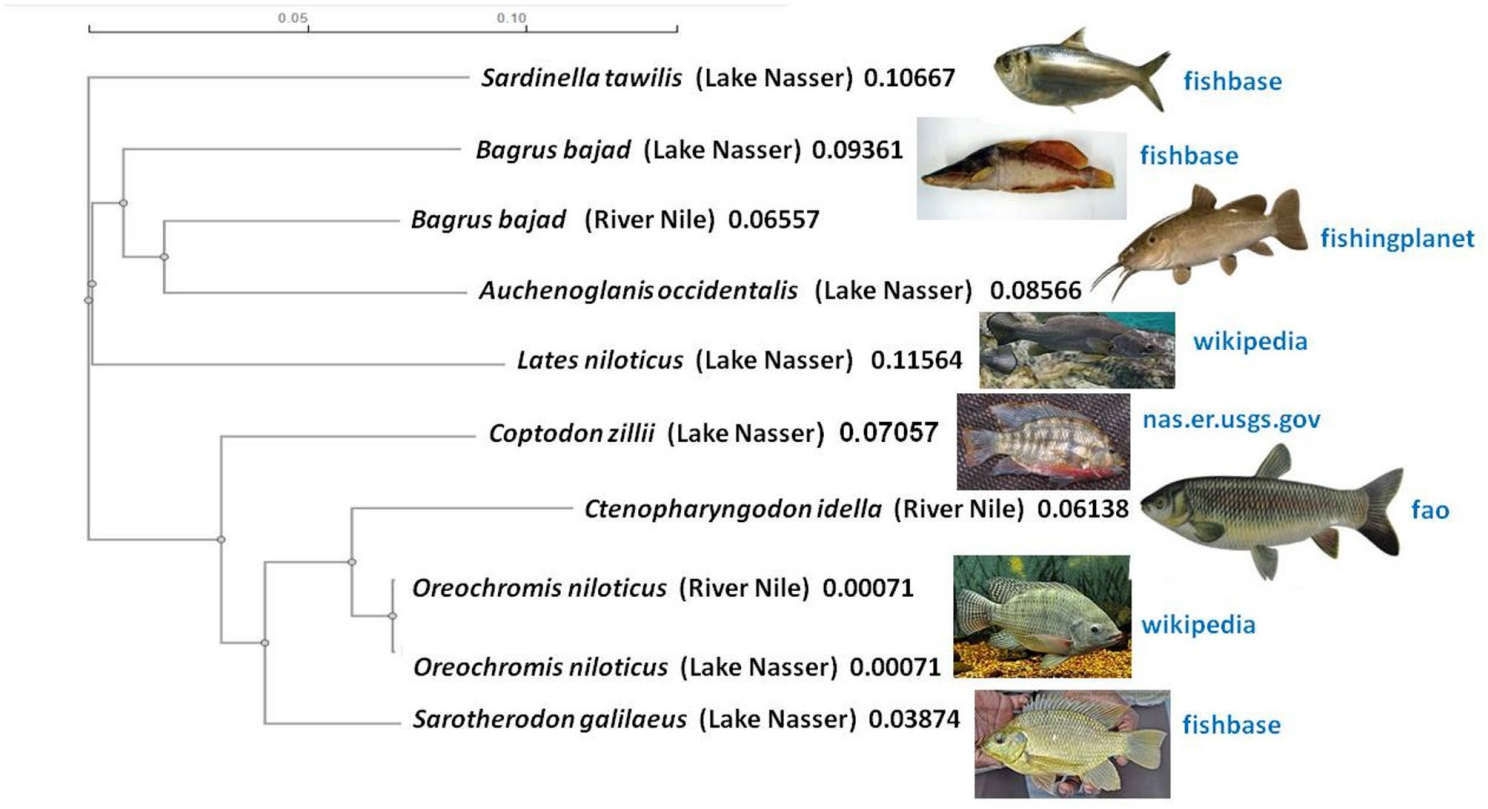
Phylogenetic tree of ten representative freshwater fish samples from Lake Nasser and the River Nile prepared using Clustal Omega with standard parameters (online version). The source for each image was indicated next to the pictures.

The COI gene sequences of the species studied from Lake Nasser and the River Nile was aligned using the Clustal Omega website (Supplementary, Figs. S1–S9) to perform the phylogenetic analysis. From the COI gene sequence alignments, there were genetic variations at a nucleotide level (A, T, C, or G) as determined at different positions of the representative sequences. The current study’s nucleotide sequence data from the COI gene of different fish samples generated an alignment of ~ 700 bp. The fish species nucleotide discrimination revealed varied AT (adenine+thiamine) and GC (guanine+cytosine) contents. Among the studied fish samples, the observed nucleotide base composition of all analyzed sequences was 53.12% AT (range: 336–388) and 46.88% GC (range: 288–356) (Table 3). The intraspecific and interspecies genetic distances were calculated with The Kimura-2-parameter model (K2P) to trace the evolutionary relationship between species, families, and orders. The K2P genetic distances between species, families, and orders from Lake Nasser and the River Nile are summarized in Tables 4, 5, 6, 7, 8, 9. The minimum genetic distance between fish samples was 0.001 (between *Oreochromis niloticus* (Lake Nasser) and *Oreochromis niloticus* (River Nile)) and the maximum distance was 0.300 (between *Lates niloticus* (Lake Nasser) and *Ctenopharyngodon idella* (River Nile)) (Table 4). The overall mean distance among fish samples was 0.20. The minimum value of genetic distance between fish families was 0.140 (between Cichlidae and Cyprinidae) and the maximum value was 0.313 (between Cyprinidae and Latidae) (Tables 6 and 8). The minimum value of genetic distance between fish orders was 0.140 (between Cichliformes and Cypriniformes) and the maximum value was 0.313 (between Perciformes and Cypriniformes) (Tables 7 and 8). The average genetic distance based on the K2P within species, families, and orders, were 0.227, 0.249, and 0.251, respectively. The minimum intraspecific genetic distance between *Oreochromis niloticus* was 0.000 and the maximum distance was 0.004 (Table 9A). The overall mean distance among *Oreochromis niloticus* within each locations was 0.00. The minimum intraspecific genetic distance between *Bagrus bajad* was 0.000 and the maximum distance was 0.209 (Table 9B). The overall mean distance among *Bagrus bajad* within each locations was 0.13. The phylogenetic trees of ten representative freshwater fish samples from Lake Nasser and the River Nile prepared using MEGA version 12.0 and Clustal Omega (online version) are shown in Figs. 2 and 3. The evolution of phylogenetic tree for the most fish species combined showed a clear distinction of the most fish families with most species of the same family were clustered together with observation of misplacement of species (Figs. 2 and 3).

**Table 4:** Estimates of pairwise genetic distances under Kimura-2-parameter distance model (K2P); in % differences for the cytochrome oxidase subunit I (COI) gene sequence between ten representative freshwater fish samples from Lake Nasser and the River Nile.

Species		Sampling location	1	2	3	4	5	6	7	8	9
1	*Ctenopharyngodon idella*	River Nile									
2	*Oreochromis niloticus*		0.078								
3	*Bagrus bajad*		0.278	0.204							
4	*Sarotherodon galilaeus*	Lake Nasser	0.143	0.073	0.207						
5	*Auchenoglanis occidentalis*		0.294	0.226	0.172	0.239					
6	*Lates niloticus*		0.300	0.241	0.209	0.249	0.268				
7	*Bagrus bajad*		0.265	0.209	0.208	0.213	0.202	0.272			
8	*Sardinella tawilis*		0.278	0.225	0.221	0.225	0.264	0.269	0.245		
9	*Coptodon zillii*		0.212	0.137	0.249	0.126	0.260	0.267	0.227	0.259	
10	*Oreochromis niloticus*		0.076	0.001	0.204	0.073	0.226	0.241	0.209	0.226	0.139

**Table 5:** Estimates of pairwise genetic distances under Kimura-2-parameter distance model (K2P); in % differences for the cytochrome oxidase subunit I (COI) gene sequence between 8 species of 30 freshwater fish samples collected from Lake Nasser and the River Nile.

Species		1	2	3	4	5	6	7
1	*Ctenopharyngodon idella*							
2	*Oreochromis niloticus*	0.089						
3	*Bagrus bajad*	0.289	0.208					
4	*Sarotherodon galilaeus*	0.156	0.074	0.211				
5	*Auchenoglanis occidentalis*	0.312	0.227	0.187	0.238			
6	*Lates niloticus*	0.313	0.238	0.237	0.247	0.265		
7	*Sardinella tawilis*	0.292	0.226	0.233	0.227	0.263	0.266	
8	*Coptodon zillii*	0.226	0.146	0.247	0.134	0.270	0.274	0.267

**Table 6:** Estimates of pairwise genetic distances under Kimura-2-parameter distance model (K2P); in % differences for the cytochrome oxidase subunit I (COI) gene sequence between families of 30 freshwater fish samples collected from Lake Nasser and the River Nile.

Family		1	2	3	4	5
1	Cyprinidae					
2	Cichlidae	0.140				
3	Bagridae	0.289	0.218			
4	Claroteidae	0.312	0.240	0.187		
5	Latidae	0.313	0.249	0.236	0.265	
6	Clupeidae	0.292	0.236	0.233	0.263	0.266

**Table 7:** Estimates of pairwise genetic distances under Kimura-2-parameter distance model (K2P); in % differences for the cytochrome oxidase subunit I (COI) gene sequence between orders of 30 freshwater fish samples collected from Lake Nasser and the River Nile.

Order		1	2	3	4
1	Cypriniformes				
2	Cichliformes	0.140			
3	Siluriformes	0.296	0.226		
4	Perciformes	0.313	0.249	0.246	
5	Clupeiformes	0.292	0.236	0.243	0.266

**Table 8:** Summary of genetic distances under Kimura-2-parameter distance model (K2P); in % differences for the cytochrome oxidase subunit I (COI) gene sequence within various taxonomic levels.

Comparisons within	Distance			Standard error
	Mean	Minimum	Maximum	
Species	0.227	0.089	0.313	0.060
Families	0.249	0.140	0.313	0.046
Orders	0.251	0.140	0.313	0.048

**Table 9:** Estimates of pairwise intraspecific genetic distances under Kimura-2-parameter distance model (K2P); in % differences for the cytochrome oxidase subunit I (COI) gene sequence within same species from Lake Nasser and the River Nile. A) for *Oreochromis niloticus,* and B) for *Bagrus bajad*.

Species				No	Sampling location		1		2		3		4		5
(A)															
1	*Oreochromis niloticus*			1	River Nile										
2	*Oreochromis niloticus*			2			0.004								
3	*Oreochromis niloticus*			3			0.000		0.001						
4	*Oreochromis niloticus*			1	Lake Nasser		0.004		0.003		0.004				
5	*Oreochromis niloticus*			2			0.001		0.000		0.001		0.003		
6	*Oreochromis niloticus*			3			0.001		0.000		0.001		0.003		0.000

Species				No	Sampling location		1		2		3		4		5
(B)															
1	*Bagrus bajad*			1	River Nile										
2	*Bagrus bajad*			2			0.000								
3	*Bagrus bajad*			3			0.002		0.002						
4	*Bagrus bajad*			1	Lake Nasser		0.208		0.208		0.206				
5	*Bagrus bajad*			2			0.208		0.208		0.206		0.000		
6	*Bagrus bajad*			3			0.209		0.209		0.207		0.000		0.000

## Discussion

Utilizing the COI gene sequence as a fish species identification method has proven to be effective in identifying fish species. This method makes it possible to standardise and informatize species identification, overcoming the over-reliance on the individual skills and experiences of taxonomists in conventional morphological classification. In the current study, we successfully amplified the COI barcode sequences for 8 fish species (3 fish species obtained from Lake Nasser and 7 fish species obtained from the River Nile). Comparison of COI gene sequences with sequences published in GenBank and BOLD revealed 99.29–100% identities at the species level. Therefore, the COI barcodes accurately identified all the studied freshwater fishes. Additionally, the COI barcode demonstrated the evolutionary linkages and interspecific genetic diversity of this group under study. The COI gene sequence showed that the DNA barcode is a useful tool to identify the fish^29–31^.

In the current study, for the 30 individuals that successfully produced COI barcodes, Records for *Ctenopharyngodon idella*, *Bagrus bajad* from Lake Nasser and the River Nile, and *Sardinella tawilis* conflicted between the molecular and morphological identification. Grass carp, *Ctenopharyngodon idella*, was yet to be barcoded in BOLD database, whereas both Bayad catfish, *Bagrus bajad*, and Bombon sardine, *Sardinella tawilis*, lacked any record in both databases. On the other hand, our results brought into question the validity of *Schilbe intermedius*, *Chrysichthys auratus*, and *Alestes baremoze* in both databases and may represent incorrect identification of the original specimen. The prevalence of this kind of inaccurate placement in early-release records shows that new specimen records should be carefully examined before being included to a database, demonstrating the continued importance of morphological identification. Therefore, morphological taxonomy, correct species, labeling, and voucher documentation should be carefully examined in case that reassessment of spurious data is required^25^. All freshwater fish species collected from Lake Nasser and the River Nile were assigned to existing BINs from the BOLD database with high accuracy which was evident in identifying species best match. Even though species identification makes extensive use of the BOLD database, however, the frailer to obtain BINs for fish species is most likely related to the lack of existing COI barcodes^21^*.* Five of nine fish species of catfish matched the reference sequences of COI gene in GenBank and the BOLD^32^.

Our results revealed that the average nucleotide base content was 53.12% AT and 46.88% GC. This result is consistent with previous studies that reported higher AT content than GC content with COI gene amplification in fish species^33–37^. Nucleotide frequencies for COI sequences gene for 44 freshwater fish sampled obtained from Enugu and Anambra States were A (24.82%), T (29.40%), C (17.70%), G (18.04%) and A+T (54.22%)^38^. The average GC% content of the 43 fish species obtained from Bay, China Rongcheng was 47.3%^37^ and the average GC% content of the 41 fish species obtained from Beas River, India was 45.47%^36^.

The highest genetic distance obtained from the COI gene sequences was 0.300% and this was 300-fold higher between ten representative freshwater fish samples than the lowest genetic distance obtained within the species (0.001%). This result is in agreement with the earlier report where genetic distance within the species is less than the one obtained from among them^21,39^. 37 fish species from the River Nile and Lake Nasser have average genetic divergence of 0.175, 0.067, and 0.008 within orders, families, and species, respectively^21^. Additionally, K2P values for 43 Indian freshwater fish species in the Beas River, India, were reported to be 0.80%, 9.06%, and 15.35% within species, genus, and families, respectively^37^. Also, K2P values for fish species in Rongcheng Bay, China, were reported to be 0.21%, 21.30%, and 23.63% within species, families, and orders, respectively^36^. The average interspecific genetic distances in Discogobio species were about 18.8 times the average intraspecific genetic distances^40^. Our results showed that genetic distances varied considerably among families and orders, and ranged from 0.140 to 0.313. The K2P values for 8 freshwater fish species in the Lake Nasser and River Nile were reported to be 0.227%, 0.249%, and 0.251% between species, families, and orders, respectively. The number of species sampled in each family may have contributed to this wide range.

The dendrogram of the studied fish species revealed by the Neighbour-joining tree and Clustal Omega showed the *Oreochromis niloticus* from Lake Nasser and the River Nile, *Sarotherodon galilaeus*, and *Coptodon zilli* clustered into one branch in the tree, whereas *Bagrus bajad* from the River Nile clustered with *Auchenoglanis occidentalis* despite their being of different family. *Bagrus bajad* from the River Nile was not clustered with *Bagrus bajad* from Lake Nasser, this supported by their sequences and the high intraspecific genetic diversity within the same species. The intra-species genetic divergence exceeding 0.209% was observed in *Bagrus bajad*, this result confirmed that the morphological identification among close fish species remains crucial. In addition, the COI barcode could uncover the morphological overlapping of the similar species. Most of the studied fish species were observed as sister group to each other at a relatively high bootstrap percentage of > 52%.

Although the tree was able to distinguish 6 families of fish, our findings showed that there were certain cases of mispositioned species even though the majority of the species in each family clustered together. This suggests a degree of evolutionary anomaly or that the selected genetic markers might not accurately reflect the relationships or morphological features of the family, even though the overall analysis shows a general evolutionary structure. A few species did not group with their closest relatives. Closely related species may be difficult to identify because to differences between morphological and COI barcoding, which could be caused by convergent evolution or cryptic diversity. It can be difficult to differentiate between species with strikingly similar features. DNA barcoding data may be highly misleading about biodiversity^41^. Allison et al.^42^ reported that the DNA barcoding is currently unreliable for identifying many crayfish species.

In conclusion, DNA barcoding and phylogenetic analysis are supper techniques for identifying fish samples regardless of sample source by analyzing short DNA sequences. This study will be helpful in Egypt and the other Nile basin countries for future researchers. It also contributes significantly to the biodiversity, conservation, and management of Lake Nasser and the River Nile by providing critical information on fish populations and the effectiveness of existing practices. In the end, this evidence can guide sustainable practices for Egyptian freshwater ecosystems by identifying threatened species, highlighting the effects of pollution and fishing gear, and demonstrating the significance of local community involvement and policy changes.

## Materials and methods

### Ethics approval

All experimental protocols used in this experiment were approved by the Committee of Alexandria University Institutional Animal Care and Use Committee (AU-IACUC) (Reference number: 19/24/05/14/1/32) before the experiment was initiated, and followed Association for Assessment and Accreditation of Laboratory Animal Care (AAALAC) protocols and guidelines. This study was carried out in compliance with the ARRIVE guidelines 2.0 (Animal Research: Reporting of In Vivo Experiment) and The American Veterinary Medical Association (AVMA) Guidelines for the Euthanasia of Animals (2020)^43^. All methods were performed in accordance with the Guide for the Care and Use of Laboratory Animals^44^, and institutional guidelines and regulations.

### Study location and sampling

**Fig. 4:**
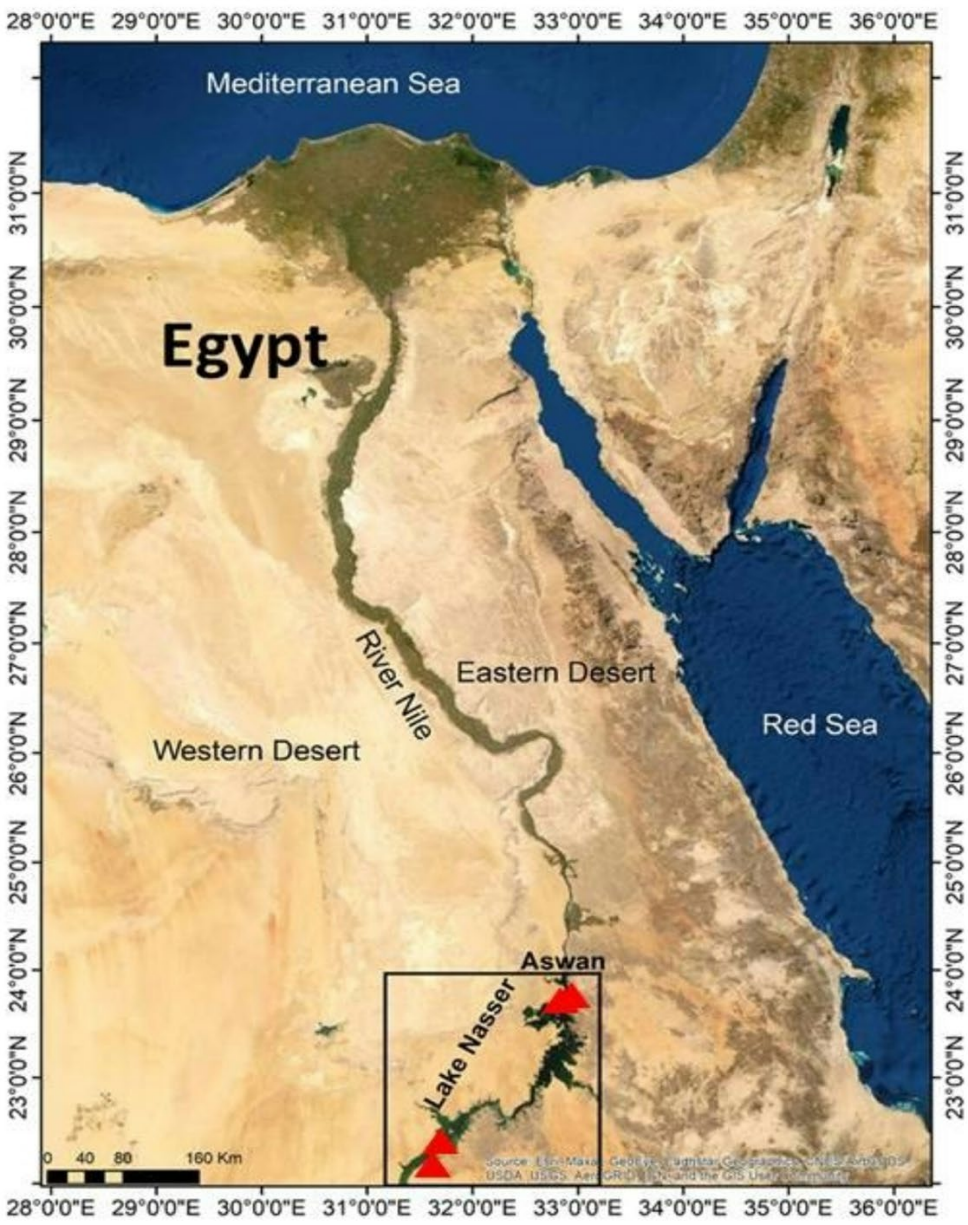
Sampling sites at Lake Nasser and River Nile in Egypt. Source: https://link.springer.com/article/10.1007/s10499-022-00839-1/figures/1.

Sampling was carried out in Aswan governorate, Egypt. The fish samples were sampled from two different locations. The two different locations were: Lake Nasser (south of the High Dam) and the River Nile (Aswan in the South) (Fig. 4).

### Fish sample and morphological identification

A total number of 30 samples were collected from two different locations in Lake Nasser (south of the High Dam) and the River Nile from Aswan in the South (Fig. 4; Table 1), in June 2024. The samples were collected by local fishermen using commercial fishing gears (nets and baskets). Fish samples were identified by their morphological characteristics^45,46^ as Grass carp *Ctenopharyngodon idella*, Nile tilapia *Oreochromis niloticus*, Bayad catfish *Bagrus bajad*, Mango tilapia *Sarotherodon galilaeus*, Yellow giraffe catfish *Auchenoglanis occidentalis*, Nile perch *Lates niloticus*, Bombon sardine *Sardinella tawilis*, and Tilapia zillii *Coptodon zillii*.

### DNA extraction

Genomic DNA was extracted from muscle tissue samples. 200 mg from each sample was placed into a 1.5-ml microcentrifuge tube containing digestion buffer followed by protein precipitation and DNA ethanol precipitation as described in the protocols of Kurita et al.^47^ and Abass et al.^48^ with some modifications. The concentration and purity of isolated DNA were confirmed on an ethidium bromide 1.2% agarose gels and NanoDrop 2000|2000c Spectrophotometers (Manufactured by ThermoScientific™, USA)^49,50^. All isolated DNA had an A260/280 ratio greater than 1.8 and were diluted to 200 ng^51^.

### Cytochrome oxidase subunit I (COI) amplification

In order to amplify ~ 700 bp fragment from the 5’ end of mitochondrial COI gene. PCR amplified using one of two sets of forward and reverse primer cocktails (Table 10)^21,25^. The amplification reactions were performed in a total volume of 10 ml and included 16 Invitrogen Platinum *Taq* Buffer, 0.25 mM each of deoxynucleotide triphosphate (dNTPs), 2.0 mM MgCl_2_,10 pmol of each primers, 100 ng of genomic DNA, and 0.5 units of *Taq* DNA polymerase. The reactions were conducted using a Thermal Cycler (Bio-Rad Laboratories, Inc., CA, USA) under the following conditions: an initial denaturation at 95 °C for 2 min; 40 cycles of 94 °C for 30 s, 62 °C for 40 s and 72 °C for 1 min; and concluded with a final elongation step of 72 °C for 10 min followed by a hold at 4 °C as described in the protocols of Abass et al.^52^. To ensure that the reactions yielded adequate amplicon sizes, PCR products were visualized and electrophoresed on a 1.5% agarose gels, containing ethidium bromide (10 mg/ml).

**Table 10:** Primer sequences for identifying the cytochrome oxidase subunit I (COI) gene from mitochondrial DNA.

Primer name	Primer direction^a^	Primer sequence (from 5′ to 3′)	Target region	Reference
COI Fish-F	F	TTC TCA ACT AAC CAY AAA GAY ATY GG	Cytochrome oxidase subunit I (COI) gene	Ali et al., 2020^21^
COI Fish-R	R	TAG ACT TCT GGG TGG CCR AAR AAY CA		
FishF1	F	TCA ACC AAC CAC AAA GAC ATT GGC AC		Ward et al., 2005^25^
FishR1	R	TAG ACT TCT GGG TGG CCA AAG AAT CA		

^a^F =  forward primer, and R = reverse primer.

### Sequencing

The Qiagen PCR Product Extraction Kit (cat# 28104) was used to purify amplified PCR products. A total of 200 ng/μl of amplified product from the 30 freshwater fish samples from Lake Nasser and the River Nile was used for sequencing with the COI Fish-F primer. All sequencing was done by the macrogen sequencing lab using the Life Technologies POP7 technology. The Abass et al.^50,52^ techniques were followed in the purification and sequencing of the amplified PCR products. Sequences from the studied fish species were manually assembled using Vector NTI software (Invitrogen Inc., CA, USA) and were submitted to the GenBank Barcode database with accession numbers PX255353-PX255382 (Table 11).

**Table 11:** List of fish species in this study and their GenBank Accession Numbers.

Species studied	No	Sampling location	GenBank Accession Number
*Ctenopharyngodon idella*	1	River Nile	PX255353
	2		PX255354
	3		PX255355
*Oreochromis niloticus*	1		PX255356
	2		PX255357
	3		PX255358
*Bagrus bajad*	1		PX255359
	2		PX255360
	3		PX255361
*Sarotherodon galilaeus*	1	Lake Nasser	PX255362
	2		PX255363
	3		PX255364
*Auchenoglanis occidentalis*	1		PX255365
	2		PX255366
	3		PX255367
*Lates niloticus*	1		PX255368
	2		PX255369
	3		PX255370
*Bagrus bajad*	1		PX255371
	2		PX255372
	3		PX255373
*Sardinella tawilis*	1		PX255374
	2		PX255375
	3		PX255376
*Coptodon zillii*	1		PX255377
	2		PX255378
	3		PX255379
*Oreochromis niloticus*	1		PX255380
	2		PX255381
	3		PX255382

### Molecular and bioinformatics data analysis

Sample identification based on the COI barcode sequence similarity approach was carried out using two databases; GenBank and the Barcode of Life Data System (BOLD), https://v3.boldsystems.org. The highest percent pairwise identity of the consensus sequence from each species blasted (BLASTN) against NCBI were compared to the percent specimen similarity scores of the consensus sequence from each species using FASTA sequences within the BOLD, accessed on September. 3^rd^, 2025. The BLASTN software available from NCBI (http://www.ncbi.nlm.nih.gov) was used to analyze DNA sequences. The Clustal Omega website (https://www.ebi.ac.uk/jdispatcher/msa/clustalo) was used to align multiple DNA sequences^52^. The phylogenetic trees were reconstructed using MEGA version 12.0^53^ and the Clustal Omega website. Phylogenetic analyses of the studied species were performed based on Neighbor-Joining (NJ) and maximum parsimony (MP) methods^54^. The robustness of the tree was assessed by performing bootstrapping analysis with 1000 replicate^55^. The Kimura-2-parameter distance model (K2P)^56^ was used to estimate the distance of the COI nucleotide bases between the fish species, families, and orders.

### Statistical analysis

The study used IBM SPSS software version 27 for all statistical analyses. Data were presented as Mean ± SD for all experiments, and subjected to one-way ANOVA, significant difference were assessed using Duncan’s^57^ multiple comparison test at *P* < 0.05.

## Supplementary Information

Below is the link to the electronic supplementary material.

Supplementary Material 1

## Data Availability

The datasets generated and analysed during the current study were deposited into the GenBank Barcode database under accession numbers PX255353-PX255382 and are available at the following URL: https://www.ncbi.nlm.nih.gov/nuccore/PX255353.1/-https://www.ncbi.nlm.nih.gov/nuccore/PX255382.

## References

[1] Society, N. G. Nile River. https://education.nationalgeographic.org/resource/nile-river/ (2025).

[2] El-Naggar, H. A., Khalaf Allah, H. M. M., Masood, M. F., Shaban, W. M. & Bashar, M. A. E. Food and feeding habits of some Nile River fish and their relationship to the availability of natural food resources. *Egypt. J. Aquat. Res.* **45**, 273–280 (2019).

[3] Alne-Na-Ei, A. A. A study of some ecological and biological characteristics of *Labeo* and *Barbus* species in Bahr Shebeen canal. Diss. Ph. D., Thesis, Zool. Dep., Fac. Sci, Menoufiya University (1994).

[4] Abdelsalam, K. M. et al. The Egyptian Nile estuarine habitats: A review. *Aquat. Sci.* **86**, 95 (2024).

[5] El Sayed, S. M., Hegab, M. H., Mola, H. R. A., Ahmed, N. M. & Goher, M. E. An integrated water quality assessment of Damietta and Rosetta branches (Nile River, Egypt) using chemical and biological indices. *Environ. Monit. Assess.* **192**, 228 (2020). 32162005 10.1007/s10661-020-8195-4

[6] Hamza, W. The Nile fishes and fisheries. in *Biodiversity—The Dynamic Balance of the Planet* (InTech, 2014). 10.5772/57381.

[7] Reid, G. M., Contreras MacBeath, T. & Csatádi, K. Global challenges in freshwater-fish conservation related to public aquariums and the aquarium industry. *Int. Zoo Yearb.* **47**, 6–45 (2013).

[8] Nwani, C. D. et al. DNA barcoding discriminates freshwater fishes from southeastern Nigeria and provides river system-level phylogeographic resolution within some species. *Mitochondrial DNA* **22**, 43–51 (2011). 21406042 10.3109/19401736.2010.536537

[9] Swartz, E. R., Mwale, M. & Hanner, R. A role for barcoding in the study of African fish diversity and conservation. *S. Afr. J. Sci.* **104**, 293–298 (2008).

[10] Van Zwieten, P. A. M., Béné, C., Kolding, J., Brummett, R. & Valbo-Jørgensen, J. E. Review of tropical reservoirs and their fisheries—The cases of Lake Nasser, Lake Volta and Indo-Gangetic Basin reservoirs. FAO Fisheries and Aquaculture Technical Paper. No. 557. 148 (Rome, FAO, 2011).

[11] GAFRD. General authority for fish resources development bulletin. in *Yearbook of fisheries statistics of Egypt*. 102 (Ministry of Agriculture and Land Reclamation, Cairo, Egypt).

[12] Goher, M. E. et al. Comprehensive insight into Lake Nasser environment: Water quality and biotic communities—a case study before operating the Renaissance Dam. *Water* **13**, 2195 (2021).

[13] Mehanna, S. F. The sustainability of Egyptian Nile River fisheries. in *The Nile River System, Africa* 197–211 (Elsevier, 2024). 10.1016/B978-0-323-90122-2.00015-2.

[14] Khalifa, U., Agaypi, M. & Adam, H. Population dynamics of *Oreochromis niloticus* L. and *Sarotherodon galilaeus*. in *Sustainable fish production in Lake Nasser: Ecological basis and management policy *(ed. Craig, J. F.) 87–90 (ICLARM, 2000).

[15] Minich, J. J. et al. Host biology, ecology and the environment influence microbial biomass and diversity in 101 marine fish species. *Nat. Commun.* **13**, 6978 (2022). 36396943 10.1038/s41467-022-34557-2 PMC9671965

[16] Zhang, J. & Hanner, R. Molecular approach to the identification of fish in the south China Sea. *PLoS ONE* **7**, e30621 (2012). 22363454 10.1371/journal.pone.0030621 PMC3281855

[17] Kim, P. S. et al. Host habitat is the major determinant of the gut microbiome of fish. *Microbiome* **9**, 166 (2021). 34332628 10.1186/s40168-021-01113-x PMC8325807

[18] FAO. Fish identification tools for biodiversity and fisheries assessments. https://www.fao.org/4/i3354e/i3354e.pdf (2013).

[19] Santanumurti, M. B. et al. Fish diversity assessment through conventional morphological identification and recent advances in Saudi Arabia: A review. *Vet. World.*10.14202/vetworld.2024.2267-2285 (2024). 39619925 10.14202/vetworld.2024.2267-2285 PMC11606295

[20] Victor, B. C., Hanner, R., Shivji, M., Hyde, J. & Caldow, C. Identification of the larval and juvenile stages of the *Cubera Snapper*, *Lutjanus cyanopterus*, using DNA barcoding. *Zootaxa* **2215**, 24–36 (2009).

[21] Ali, F. S., Ismail, M. & Aly, W. DNA barcoding to characterize biodiversity of freshwater fishes of Egypt. *Mol. Biol. Rep.* **47**, 5865–5877 (2020). 32661870 10.1007/s11033-020-05657-3

[22] Hamza, A. M. & Mohammed, A. T. E. M. DNA barcoding of five economically important freshwater fish species from the Nile River. *Sudan. African J. Aquat. Sci.* **48**, 338–343 (2023).

[23] Costa, F. O. & Antunes, P. M. The contribution of the barcode of life initiative to the discovery and monitoring of biodiversity. *Nat. Resour. Sustain. Humanit.*10.1007/978-94-007-1321-5_4 (2012).

[24] Elsaied, H., Soliman, T., Abdelmageed, A. A. & Abu-Taleb, H. T. Applications and challenges of DNA barcoding and metabarcoding in African fisheries. *Egypt. J. Aquat. Res.* **47**, 1–12 (2021).

[25] Ward, R. D., Zemlak, T. S., Innes, B. H., Last, P. R. & Hebert, P. D. DNA barcoding Australia’s fish species. *Philos. Trans. R. Soc. B Biol. Sci.* **360**, 1847–1857 (2005). 10.1098/rstb.2005.1716 PMC1609232 16214743

[26] Hebert, P. D. N., Ratnasingham, S. & de Waard, J. R. Barcoding animal life: Cytochrome c oxidase subunit 1 divergences among closely related species. *Proc. R. Soc. London. Ser. B Biol. Sci.* **270**, 96–99 (2003). 10.1098/rsbl.2003.0025 PMC1698023 12952648

[27] Nwani, C. D. et al. DNA barcoding discriminates freshwater fishes from southeastern Nigeria and provides river system-level phylogeographic resolution within some species. *Mitochondrial DNA* **22**, 43–51 (2011). 21406042 10.3109/19401736.2010.536537

[28] Froese, R. and D. P. FishBase. World Wide Web electronic publication. http://www.fishbase.org, version (04/2025) (2025).

[29] Bingpeng, X. et al. DNA barcoding for identification of fish species in the Taiwan Strait. *PLoS ONE* **13**, e0198109 (2018). 29856794 10.1371/journal.pone.0198109 PMC5983523

[30] Chang, C. et al. DNA barcodes of the native ray-finned fishes in Taiwan. *Mol. Ecol. Resour.* **17**, 796–805 (2017). 27717215 10.1111/1755-0998.12601

[31] Ahmed, M. S., Datta, S. K., Saha, T. & Hossain, Z. Molecular characterization of marine and coastal fishes of Bangladesh through DNA barcodes. *Ecol. Evol.* **11**, 3696–3709 (2021). 33976769 10.1002/ece3.7355 PMC8093680

[32] Wong, L. L. et al. DNA Barcoding of catfish: Species authentication and phylogenetic assessment. *PLoS ONE* **6**, e17812 (2011). 21423623 10.1371/journal.pone.0017812 PMC3057997

[33] Ghouri, M. Z. et al. Identification of edible fish species of Pakistan through DNA barcoding. *Front. Mar. Sci.* **7**, 554183 (2020).

[34] Nei, M. & Kumar, S. *Molecular Evolution and Phylogenetics* (Oxford University Press, 2000).

[35] Sajjad, A., Jabeen, F., Ali, M. & Zafar, S. DNA barcoding and phylogenetics of *Wallago attu* using mitochondrial COI gene from the River Indus. *J. King Saud Univ. - Sci.* **35**, 102725 (2023).

[36] Modeel, S. et al. A comprehensive DNA barcoding of Indian freshwater fishes of the Indus River system. *Beas. Sci. Rep.* **14**, 2763 (2024). 38307873 10.1038/s41598-024-52519-0 PMC10837433

[37] Wang, L. et al. DNA barcoding of marine fish species from Rongcheng Bay, China. *PeerJ* **6**, e5013 (2018). 29967722 10.7717/peerj.5013 PMC6022726

[38] Ude, G. N. et al. DNA barcoding for identification of fish species from freshwater in Enugu and Anambra States of Nigeria. *Conserv. Genet. Resour.* **12**, 643–658 (2020).

[39] Martinez, A. S., Willoughby, J. R. & Christie, M. R. Genetic diversity in fishes is influenced by habitat type and life-history variation. *Ecol. Evol.* **8**, 12022–12031 (2018). 30598796 10.1002/ece3.4661 PMC6303716

[40] Li, H., Cheng, H., Huang, R., Qiu, Z. & Zhang, R. DNA barcoding of the genus *Discogobio* (Teleostei, Cyprinidae) in China. *Fishes* **10**, 157 (2025).

[41] Trewick, S. A. DNA Barcoding is not enough: Mismatch of taxonomy and genealogy in New Zealand grasshoppers (Orthoptera: Acrididae). *Cladistics* **24**, 240–254 (2008).

[42] Allison, P. F., Pickich, E. T., Barnett, Z. C. & Garrick, R. C. DNA barcoding is currently unreliable for species identification in most crayfishes. *Ecol. Evol.* **14**, e70050 (2024). 39041008 10.1002/ece3.70050 PMC11260883

[43] AVMA guidelines for the euthanasia of animals: 2020 edition. https://www.avma.org/sites/default/files/2020-02/Guidelines-on-Euthanasia-2020.pdf.

[44] Kilkenny, C., Browne, W. J., Cuthill, I. C., Emerson, M. & Altman, D. G. Improving bioscience research reporting: The ARRIVE Guidelines for Reporting Animal Research. *PLoS Biol.* **8**, e1000412 (2010). 20613859 10.1371/journal.pbio.1000412 PMC2893951

[45] Bishai, H. M. & Khalil, M. T. Freshwater fishes of Egypt*.* Vol. 9. 1–229 (Publication of National Biodiversity Unit, 1997).

[46] Froese, R., & Pauly, D. Eds. FishBase. World Wide Web electronicpublication. https://www.fishbase.org, version. (2016).

[47] Kurita, K., Burgess, S. M. & Sakai, N. Transgenic zebrafish produced by retroviral infection of in vitro-cultured sperm. *Proc. Natl. Acad. Sci.* **101**, 1263–1267 (2004). 14745028 10.1073/pnas.0304265101 PMC337041

[48] Abass, N. Y. et al. Effects of family and promoter on growth performance of ccGH cDNA transgenic channel catfish, *Ictalurus punctatus*, grown in a trough culture system. *Aquaculture* **536**, 736468 (2021).

[49] Abass, N. Y. et al. Genotype-environment interactions for survival and growth rate at varying levels of sodium chloride for growth hormone transgenic channel catfish (*Ictalurus punctatus*), channel catfish, and albino channel catfish. *Aquaculture* **521**, 735084 (2020).

[50] Abass, N. Y. et al. Response of cecropin transgenesis to challenge with *Edwardsiella ictaluri* in channel catfish *Ictalurus punctatus*. *Fish Shellfish Immunol.* **126**, 311–317 (2022). 35636698 10.1016/j.fsi.2022.05.050

[51] Abass, N. Y., Ye, Z., Alsaqufi, A. & Dunham, R. A. Comparison of growth performance among channel-blue hybrid catfish, ccGH transgenic channel catfish, and channel catfish in a tank culture system. *Sci. Rep.* **12**, 740 (2022). 35031641 10.1038/s41598-021-04719-1 PMC8760261

[52] Abass, N. Y. et al. Genotype–environment interactions for survival at low and sub-zero temperatures at varying salinity for channel catfish, hybrid catfish and transgenic channel catfish. *Aquaculture* **458**, 140–148 (2016).

[53] Kumar, S. et al. MEGA12: Molecular evolutionary genetic analysis version 12 for adaptive and green computing. *Mol. Biol. Evol.* **41**, 12 (2024). 10.1093/molbev/msae263 PMC11683415 39708372

[54] Nei, M. & Kumar, S. *Molecular Evolution and Phylogenetics* (Oxford University Press, New York, 2000).

[55] Felsenstein, J. Confidence limits on phylogenies: An approach using the bootstrap. *Evolution (NY).* **39**, 783–791 (1985). 10.1111/j.1558-5646.1985.tb00420.x 28561359

[56] Kimura, M. A simple method for estimating evolutionary rates of base substitutions through comparative studies of nucleotide sequences. *J. Mol. Evol.* **16**, 111–120 (1980). 7463489 10.1007/BF01731581

[57] Duncan, D. B. Multiple range and multiple *F* tests. *Biometrics* **11**, 1 (1955).

