# Supplementary material for: DNA barcoding and phylogenetic analysis to characterize biodiversity of some freshwater fish species in Lake Nasser and River Nile
Source: Sci Rep. 2025 Oct 9;15:35237. doi: 10.1038/s41598-025-20830-z (PMC12511596; doi:10.1038/s41598-025-20830-z)
Supplement: Supplementary file 1 — Supplementary Material 1 [file 41598_2025_20830_MOESM1_ESM.docx]

**DNA barcoding and phylogenetic analysis to characterize biodiversity of some freshwater fish species in Lake Nasser and River Nile**

Esraa Mostafa Ashour^a^, Ragaa A. Ahmed^b^, & Nermeen Y. Abass^a*^

^a^ Department of Agricultural Botany, Faculty of Agriculture Saba-Basha, Alexandria University, Alexandria City, P.O. Box 21531, Egypt

^b^ Department of Aquaculture, Faculty of Fish and Fisheries Technology, Aswan University, Aswan, Egypt

*Corresponding author: + 2 03 5831646; fax: + 2 035832008

E-mail address: [n.y.abass@alexu.edu.eg](mailto:n.y.abass@alexu.edu.eg)

**PX255353 TCAACCAACCACAAGGATATGGGCACCCTCTATCTAGTATTTGGTGCTTGAGCCGGAATA 60**

**PX255355 TCAACCAACCACAAGGATATGGGCACCCTCTATCTAGTATTTGGTGCTTGAGCCGGAATA 60**

**PX255354 TCAACCAACCACAAGGATATGGGCACCCTCTATCTAGTATTTGGTGCTTGAGCCGGAATA 60**

****************************************************************

**PX255353 GTAGGAACTGCACTAAGCCTCCTAATTCGGGCAGAACTAAGCCAGCCCGGCTCTCTTCTC 120**

**PX255355 GTAGGAACTGCACTAAGCCTCCTAATTCGGGCAGAACTAAGCCAGCCCGGCTCTCTTCTC 120**

**PX255354 GTAGGAACTGCACTAAGCCTCCTAATTCGGGCAGAACTAAGCCAGCCCGGCTCTCTTCTC 120**

****************************************************************

**PX255353 GGAGACGACCAAATCTATAATGTAATTGTTACAGCACATGCTTTCGTAATAATTTCCATT 180**

**PX255355 GGAGACGACCAAATCTATAATGTAATTGTTACAGCACATGCTTTCGTAATAATTTCCATT 180**

**PX255354 GGAGACGACCAAATCTATAATGTAATTGTTACAGCACATGCTTTCGTAATAATTTTCTTT 180**

********************************************************* * ****

**PX255353 ATTCCTATGTCTTTTCTGATTGCATGCTTTGGAAGTTGACAAGTTCCTTTTATGATTGGT 240**

**PX255355 ATTCCTATGTCTTTTCTGATTGCATGCTTTGGAAGTTGACAAGTTCCTTTTATGATTGGT 240**

**PX255354 ATAGTAATACCAATTATGATTGCATGCTTTGGAAGTTGACAAGTTCCTTTTATGATTGGT 240**

**** ** * ** **********************************************

**PX255353 GCAGTTGATGAGGACTTTCCTCGCATAAGCAAGAACTTTTGACTTCTCCCCCCCTCGTTT 300**

**PX255355 GCAGTTGATGAGGACTTTCCTCGCATAAGCAAGAACTTTTGACTTCTCCCCCCCTCGTTT 300**

**PX255354 GCAGTTGATGAGGACTTTCCTCGCATAAGCAAGAACTTTTGACTTCTCCCCCCCTCGTTT 300**

****************************************************************

**PX255353 CTTCATCTTCTCTCCTCTTCTTGAGTCGAAGCATGTGCCGGCACATGATGGACTGTTTAT 360**

**PX255355 CTTCATCTTCTCTCCTCTTCTTGAGTCGAAGCATGTGCCGGCACATGATGGACTGTTTAT 360**

**PX255354 CTTCATCTTCTCTCCTCTTCTTGAGTCGAAGCATGTGCCGGCACATGATGGACTGTTTAT 360**

****************************************************************

**PX255353 CCCCCGCTCCCGGGCAATCTTGCCCACGCTGGACCTTCTGTTGACTTAACCATCTTCTCC 420**

**PX255355 CCCCCGCTCCCGGGCAATCTTGCCCACGCTGGACCTTCTGTTGACTTAACCATCTTCTCC 420**

**PX255354 CCCCCGCTCCCGGGCAATCTTGCCCACGCTGGACCTTCTGTTGACTTAACCATCTTCTCC 420**

****************************************************************

**PX255353 CTCAACTTGGCCGGAATGTTATCTATTTTATGTGCTTTTAATTTTATAACAACCATTATT 480**

**PX255355 CTCAACTTGGCCGGAATGTTATCTATTTTATGTGCTTTTAATTTTATAACAACCATTATT 480**

**PX255354 CTCAACTTGGCCGGAATGTTATCTATTTTATGTGCTTTTAATTTTATAACAACCATTATT 480**

****************************************************************

**PX255353 AACATGAAACCCCCTGCCGTCTCCCAATATCAAACACCCCTATTTGTGTGATCCGTCCTA 540**

**PX255355 AACATGAAACCCCCTGCCGTCTCCCAATATCAAACACCCCTATTTGTGTGATCCGTCCTA 540**

**PX255354 AACATGAAACCCCCTGCCGTCTCCCAATATCAAACACCCCTATTTGTGTGATCCGTCCTA 540**

****************************************************************

**PX255353 ATTACCGCACTACTACTCCTTCTATCCCTGCCCGTTCTTGCCGCCGGCATCACAATACTT 600**

**PX255355 ATTACCGCACTACTACTCCTTCTATCCCTGCCCGTTCTTGCCGCCGGCATCACAATACTT 600**

**PX255354 ATTACCGCACTACTACTCCTTCTATCCCTGCCCGTTCTTGCCGCCGGCATCACAATACTT 600**

****************************************************************

**PX255353 CTAACAGACCGAAACCTTAACACTACATTCTTTGACCCGGCGGGAGGAGGAGACCCAATT 660**

**PX255355 CTAACAGACCGAAACCTTAACACTACATTCTTTGACCCGGCGGGAGGAGGAGACCCAATT 660**

**PX255354 CTAACAGACCGAAACCTTAACACTACATTCTTTGACCCGGCGGGAGGAGGAGACCCAATT 660**

****************************************************************

**PX255353 CTTTATCAACACTTATTCTGATTCTTCGGTCACCCTGAAGTGTCA 705**

**PX255355 CTTTATCAACACTTATTCTGATTCTTCGGTCACCCTGAAGTGTCA 705**

**PX255354 CTTTATCAACACTTATTCTGATTCTTCGGTCACCCTGAAGTGTCA 705**

*************************************************

**Figure S1.** Alignment of sequencing result for PCR product from three fish samples of *Ctenopharyngodon idella* from the River Nile, generated by Clustal Omega. Identities and similarities are indicated by star (*) and gaps are indicated by dashes (-).

**PX255380 TCAACCAAC---CACAAGGATATGGGCTCGTATCTAGTATTTGGTGCTTGAGCCGGAATA 57**

**PX255356 TCAACCAACCACAAGGATATTGGCACCCTCTATCTAGTATTTGGTGCTTGAGCCGGAATA 60**

**PX255358 TCAACCAACCACAAGGATATTGGCACCCTCTATCTAGTATTTGGTGCTTGAGCCGGAATA 60**

**PX255357 TCAACCAACCACAAGGATATGGGCACCCTCTATCTAGTATTTGGTGCTTGAGCCGGAATA 60**

**PX255381 TCAACCAACCACAAGGATATGGGCACCCTCTATCTAGTATTTGGTGCTTGAGCCGGAATA 60**

**PX255382 TCAACCAACCACAAGGATATGGGCACCCTCTATCTAGTATTTGGTGCTTGAGCCGGAATA 60**

*********** * * * ********************************

**PX255380 GTAGGAACTGCACTAAGCCTCCTAATTCGGGCAGAACTAAGCCAGCCCGGCTCTCTTCTC 117**

**PX255356 GTAGGAACTGCACTAAGCCTCCTAATTCGGGCAGAACTAAGCCAGCCCGGCTCTCTTCTC 120**

**PX255358 GTAGGAACTGCACTAAGCCTCCTAATTCGGGCAGAACTAAGCCAGCCCGGCTCTCTTCTC 120**

**PX255357 GTAGGAACTGCACTAAGCCTCCTAATTCGGGCAGAACTAAGCCAGCCCGGCTCTCTTCTC 120**

**PX255381 GTAGGAACTGCACTAAGCCTCCTAATTCGGGCAGAACTAAGCCAGCCCGGCTCTCTTCTC 120**

**PX255382 GTAGGAACTGCACTAAGCCTCCTAATTCGGGCAGAACTAAGCCAGCCCGGCTCTCTTCTC 120**

****************************************************************

**PX255380 GGAGACGACCAAATCTATAATGTAATTGTTACAGCACATGCTTTCGTAATAATTTTCTTT 177**

**PX255356 GGAGACGACCAAATCTATAATGTAATTGTTACAGCACATGCTTTCGTAATAATTTTCTTT 180**

**PX255358 GGAGACGACCAAATCTATAATGTAATTGTTACAGCACATGCTTTCGTAATAATTTTCTTT 180**

**PX255357 GGAGACGACCAAATCTATAATGTAATTGTTACAGCACATGCTTTCGTAATAATTTTCTTT 180**

**PX255381 GGAGACGACCAAATCTATAATGTAATTGTTACAGCACATGCTTTCGTAATAATTTTCTTT 180**

**PX255382 GGAGACGACCAAATCTATAATGTAATTGTTACAGCACATGCTTTCGTAATAATTTTCTTT 180**

****************************************************************

**PX255380 ATAGTAATACCAATTATGATTGGAGGCTTTGGAAACTGACTAGTACCCCTCATGATTGGT 237**

**PX255356 ATAGTAATACCAATTATGATTGGAGGCTTTGGAAACTGACTAGTACCCCTCATGATTGGT 240**

**PX255358 ATAGTAATACCAATTATGATTGGAGGCTTTGGAAACTGACTAGTACCCCTCATGATTGGT 240**

**PX255357 ATAGTAATACCAATTATGATTGGAGGCTTTGGAAACTGACTAGTACCCCTCATGATTGGT 240**

**PX255381 ATAGTAATACCAATTATGATTGGAGGCTTTGGAAACTGACTAGTACCCCTCATGATTGGT 240**

**PX255382 ATAGTAATACCAATTATGATTGGAGGCTTTGGAAACTGACTAGTACCCCTCATGATTGGT 240**

****************************************************************

**PX255380 GCACCAGACATGGCCTTCCCTCGAATAAATAACATGAGCTTTTGACTTCTCCCCCCCTCA 297**

**PX255356 GCACCAGACATGGCCTTCCCTCGAATAAATAACATGAGCTTTTGACTTCTCCCCCCCTCA 300**

**PX255358 GCACCAGACATGGCCTTCCCTCGAATAAATAACATGAGCTTTTGACTTCTCCCCCCCTCA 300**

**PX255357 GCACCAGACATGGCCTTCCCTCGAATAAATAACATGAGCTTTTGACTTCTCCCCCCCTCA 300**

**PX255381 GCACCAGACATGGCCTTCCCTCGAATAAATAACATGAGCTTTTGACTTCTCCCCCCCTCA 300**

**PX255382 GCACCAGACATGGCCTTCCCTCGAATAAATAACATGAGCTTTTGACTTCTCCCCCCCTCA 300**

****************************************************************

**PX255380 TTTCTTCTTCTTCTCGCCTCATCTGGAGTCGAAGCAGGTGCCGGCACAGGATGGACTGTT 357**

**PX255356 TTTCTTCTTCTTCTCGCCTCATCTGGAGTCGAAGCAGGTGCCGGCACAGGATGGACTGTT 360**

**PX255358 TTTCTTCTTCTTCTCGCCTCATCTGGAGTCGAAGCAGGTGCCGGCACAGGATGGACTGTT 360**

**PX255357 TTTCTTCTTCTTCTCGCCTCATCTGGAGTCGAAGCAGGTGCCGGCACAGGATGGACTGTT 360**

**PX255381 TTTCTTCTTCTTCTCGCCTCATCTGGAGTCGAAGCAGGTGCCGGCACAGGATGGACTGTT 360**

**PX255382 TTTCTTCTTCTTCTCGCCTCATCTGGAGTCGAAGCAGGTGCCGGCACAGGATGGACTGTT 360**

****************************************************************

**PX255380 TATCCCCCGCTCGCAGGCAATCTTGCCCACGCTGGACCTTCTGTTGACTTAACCATCTTC 417**

**PX255356 TATCCCCCGCTCGCAGGCAATCTTGCCCACGCTGGACCTTCTGTTGACTTAACCATCTTC 420**

**PX255358 TATCCCCCGCTCGCAGGCAATCTTGCCCACGCTGGACCTTCTGTTGACTTAACCATCTTC 420**

**PX255357 TATCCCCCGCTCGCAGGCAATCTTGCCCACGCTGGACCTTCTGTTGACTTAACCATCTTC 420**

**PX255381 TATCCCCCGCTCGCAGGCAATCTTGCCCACGCTGGACCTTCTGTTGACTTAACCATCTTC 420**

**PX255382 TATCCCCCGCTCGCAGGCAATCTTGCCCACGCTGGACCTTCTGTTGACTTAACCATCTTC 420**

****************************************************************

**PX255380 TCCCTCCACTTGGCCGGAGTGTCATCTATTTTAGGTGCAATTAATTTTATCACAACCATT 477**

**PX255356 TCCCTCCACTTGGCCGGAGTGTCATCTATTTTAGGTGCAATTAATTTTATCACAACCATT 480**

**PX255358 TCCCTCCACTTGGCCGGAGTGTCATCTATTTTAGGTGCAATTAATTTTATCACAACCATT 480**

**PX255357 TCCCTCCACTTGGCCGGAGTGTCATCTATTTTAGGTGCAATTAATTTTATCACAACCATT 480**

**PX255381 TCCCTCCACTTGGCCGGAGTGTCATCTATTTTAGGTGCAATTAATTTTATCACAACCATT 480**

**PX255382 TCCCTCCACTTGGCCGGAGTGTCATCTATTTTAGGTGCAATTAATTTTATCACAACCATT 480**

****************************************************************

**PX255380 ATTAACATGAAACCCCCTGCCATCTCCCAATATCAAACACCCCTATTTGTGTGATCCGTC 537**

**PX255356 ATTAACATGAAACCCCCTGCCATCTCCCAATATCAAACACCCCTATTTGTGTGATCCGTC 540**

**PX255358 ATTAACATGAAACCCCCTGCCATCTCCCAATATCAAACACCCCTATTTGTGTGATCCGTC 540**

**PX255357 ATTAACATGAAACCCCCTGCCATCTCCCAATATCAAACACCCCTATTTGTGTGATCCGTC 540**

**PX255381 ATTAACATGAAACCCCCTGCCATCTCCCAATATCAAACACCCCTATTTGTGTGATCCGTC 540**

**PX255382 ATTAACATGAAACCCCCTGCCATCTCCCAATATCAAACACCCCTATTTGTGTGATCCGTC 540**

****************************************************************

**PX255380 CTAATTACCGCAGTACTACTCCTTCTATCCCTGCCCGTTCTTGCCGCCGGCATCACAATA 597**

**PX255356 CTAATTACCGCAGTACTACTCCTTCTATCCCTGCCCGTTCTTGCCGCCGGCATCACAATA 600**

**PX255358 CTAATTACCGCAGTACTACTCCTTCTATCCCTGCCCGTTCTTGCCGCCGGCATCACAATA 600**

**PX255357 CTAATTACCGCAGTACTACTCCTTCTATCCCTGCCCGTTCTTGCCGCCGGCATCACAATA 600**

**PX255381 CTAATTACCGCAGTACTACTCCTTCTATCCCTGCCCGTTCTTGCCGCCGGCATCACAATA 600**

**PX255382 CTAATTACCGCAGTACTACTCCTTCTATCCCTGCCCGTTCTTGCCGCCGGCATCACAATA 600**

****************************************************************

**PX255380 CTTCTAACAGACCGAAACCTAAACACAACCTTCTTTGACCCTGCCGGAGGAGGAGACCCC 657**

**PX255356 CTTCTAACAGACCGAAACCTAAACACAACCTTCTTTGACCCTGCCGGAGGAGGAGACCCC 660**

**PX255358 CTTCTAACAGACCGAAACCTAAACACAACCTTCTTTGACCCTGCCGGAGGAGGAGACCCC 660**

**PX255357 CTTCTAACAGACCGAAACCTAAACACAACCTTCTTTGACCCTGCCGGAGGAGGAGACCCC 660**

**PX255381 CTTCTAACAGACCGAAACCTAAACACAACCTTCTTTGACCCTGCCGGAGGAGGAGACCCC 660**

**PX255382 CTTCTAACAGACCGAAACCTAAACACAACCTTCTTTGACCCTGCCGGAGGAGGAGACCCC 660**

****************************************************************

**PX255380 ATCCTATACCAACACTTATTCTGATTCTTCGGCCACCCAGAAGTT 702**

**PX255356 ATCCTATACCAACACTTATTCTGATTCTTCGGCCACCCAGAAGTT 705**

**PX255358 ATCCTATACCAACACTTATTCTGATTCTTCGGCCACCCAGAAGTT 705**

**PX255357 ATCCTATACCAACACTTATTCTGATTCTTCGGCCACCCAGAAGTT 705**

**PX255381 ATCCTATACCAACACTTATTCTGATTCTTCGGCCACCCAGAAGTT 705**

**PX255382 ATCCTATACCAACACTTATTCTGATTCTTCGGCCACCCAGAAGTT 705**

*************************************************

**Figure S2.** Alignment of sequencing result for PCR product from six fish samples of *Oreochromis niloticus* from Lake Nasser and the River Nile, generated by Clustal Omega. Identities and similarities are indicated by star (*) and gaps are indicated by dashes (-).

**PX255359 ------CCTAGTATTTGGTGCCTGAGCCGGAATAGTCGGCACAGCCCTAAGCCTACTTAT 54**

**PX255360 ------CCTAGTATTTGGTGCCTGAGCCGGAATAGTCGGCACAGCCCTAAGCCTACTTAT 54**

**PX255361 -CTCTACCTAGTATTTGGTGCCTGAGCCGGAATAGTCGGCACAGCCCTAAGCCTACTTAT 59**

**PX255371 CCTTTACATAGCAATTGGTGCTTGAGCCGGCATAGTGGGTACGGCCCTCAGCCTATTAAT 60**

**PX255372 CCTTTACATAGCAATTGGTGCTTGAGCCGGCATAGTGGGTACGGCCCTCAGCCTATTAAT 60**

**PX255373 CCTTTACATAGCAATTGGTGCTTGAGCCGGCATAGTGGGTACG---GTCAGCCTATTAAT 57**

*** *** * ******* ******** ***** ** ** * ****** * ****

**PX255359 TCGGGCAGAGCTAGCCCAACCTGGCGCTCTCCTGGGAGACGACCAAATTTATAATGTTAT 114**

**PX255360 TCGGGCAGAGCTAGCCCAACCTGGCGCTCTCCTGGGAGACGACCAAATTTATAATGTTAT 114**

**PX255361 TCGGGCAGAGCTAGCCCAACCTGGCGCTCTCCTGGGAGACGACCAAATTTATAATGTTAT 119**

**PX255371 TCGGGCAGAACTAGCCCAACCCGGAGCCCTTCTGGGCGACGACCAGATTTATAATGTAAT 120**

**PX255372 TCGGGCAGAACTAGCCCAACCCGGAGCCCTTCTGGGCGACGACCAGATTTATAATGTAAT 120**

**PX255373 TCGGGCAGAACTAGCCCAACCCGGAGCCCTTCTGGGCGACGACCAGATTTATAATGTAAT 117**

*********** *********** ** ** ** ***** ******** *********** ****

**PX255359 TGTTACTGCCCATGCCTTCGTAATAATCTTCTTTATAGTAATACCAATTATGATTGGAGG 174**

**PX255360 TGTTACTGCCCATGCCTTCGTAATAATCTTCTTTATAGTAATACCAATTATGATTGGAGG 174**

**PX255361 TGTTACTGCCCATGCCTTCGTAATAATCTTCTTTATAGTAATACCAATTATGATTGGAGG 179**

**PX255371 TGTTACTGCTCATGCCTTTGTAATAATTTTCTTTATAGTAATGCCAATCATAATTGGAGG 180**

**PX255372 TGTTACTGCTCATGCCTTTGTAATAATTTTCTTTATAGTAATGCCAATCATAATTGGAGG 180**

**PX255373 TGTTACTGCTCATGCCTTTGTAATAATTTTCTTTATAGTAATGCCAATCATAATTGGAGG 177**

*********** ******** ******** ************** ***** ** **********

**PX255359 CTTTGGAAACTGACTTATCCCACTAATAATCGGAGCCCCAGACATAGCATTCCCCCGAAT 234**

**PX255360 CTTTGGAAACTGACTTATCCCACTAATAATCGGAGCCCCAGACATAGCATTCCCCCGAAT 234**

**PX255361 CTTTGGAAACTGACTTATCCCACTAATAATCGGAGCCCCAGACATAGCATTCCCCCGAAT 239**

**PX255371 CTTTGGGAACTGGCTGGTTCCACTAATGATTGGAGCACCAGACATGGCCTTTCCCCGAAT 240**

**PX255372 CTTTGGGAACTGGCTGGTTCCACTAATGATTGGAGCACCAGACATGGCCTTTCCCCGAAT 240**

**PX255373 CTTTGGGAACTGGCTGGTTCCACTAATGATTGGAGCACCAGACATGGCCTTTCCCCGAAT 237**

******** ***** ** * ******** ** ***** ******** ** ** **********

**PX255359 AAATAACATGAGCTTCTGACTACTCCCTCCCTCCTTCTTACTACTTCTAGCCTCCTCCGG 294**

**PX255360 AAATAACATGAGCTTCTGACTACTCCCTCCCTCCTTCTTACTACTTCTAGCCTCCTCCGG 294**

**PX255361 AAATAACATGAGCTTCTGACTACTCCCTCCCTCCTTCTTACTACTTCTAGCCTCCTCCGG 299**

**PX255371 AAATAACATAAGCTTCTGACTTCTCCCCCCCTCTTTCTTATTACTGCTTGCCTCATCAGG 300**

**PX255372 AAATAACATAAGCTTCTGACTTCTCCCCCCCTCTTTCTTATTACTGCTTGCCTCATCAGG 300**

**PX255373 AAATAACATAAGCTTCTGACTTCTCCCCCCCTCTTTCTTATTACTGCTTGCCTCATCAGG 297**

*********** *********** ***** ***** ****** **** ** ***** ** ****

**PX255359 AGTTGAGGCAGGAGCAGGAACAGGATGAACTGTATATCCCCCCCTCGCTGGAAACCTCGC 354**

**PX255360 AGTTGAGGCAGGAGCAGGAACAGGATGAACTGTATATCCCCCCCTCGCTGGAAACCTCGC 354**

**PX255361 AGTTGAGGCAGGAGCAGGAACAGGATGAACTGTATATCCCCCCCTCGCTGGAAACCTTGC 359**

**PX255371 AGTTGAAGCAGGGGCAGGAACAGGATGAACTGTTTACCCGCCTCTCGCCGGAAACCTTGC 360**

**PX255372 AGTTGAAGCAGGGGCAGGAACAGGATGAACTGTTTACCCGCCTCTCGCCGGAAACCTTGC 360**

**PX255373 AGTTGAAGCAGGGGCAGGAACAGGATGAACTGTTTACCCGCCTCTCGCCGGAAACCTTGC 357**

******** ***** ******************** ** ** ** ***** ******** ****

**PX255359 ACACGCCGGAGCCTCTGTAGATCTAACTATTTTCTCCCTCCACCTGGCAGGTGTATCTTC 414**

**PX255360 ACACGCCGGAGCCTCTGTAGATCTAACTATTTTCTCCCTCCACCTGGCAGGTGTATCTTC 414**

**PX255361 ACACGCCGGAGCCTCTGTAGATCTAACTATTTTCTCCCTCCACCTGGCAGGTGTATCTTC 419**

**PX255371 TCATGCGGGGGCCTCTGTAGACTTAACCATCTTTTCACTCCATCTTGCAGGTGTTTCCTC 420**

**PX255372 TCATGCGGGGGCCTCTGTAGACTTAACCATCTTTTCACTCCATCTTGCAGGTGTTTCCTC 420**

**PX255373 TCATGCGGGGGCCTCTGTAGACTTAACCATCTTTTCACTCCATCTTGCAGGTGTTTCCTC 417**

**** ** ** *********** **** ** ** ** ***** ** ******** ** ****

**PX255359 TATTCTAGGGGCCATCAACTTCATTACCACAATCATTAACATAAAACCCCCAGCCATCTC 474**

**PX255360 TATTCTAGGGGCCATCAACTTCATTACCACAATCATTAACATAAAACCCCCAGCCATCTC 474**

**PX255361 TATTCTAGGGGCCATCAACTTCATTACCACAATCATTAACATAAAACCCCCAGCCATCTC 479**

**PX255371 CATCCTGGGTGCAATCAATTTTATTACAACCATTATTAATATGAAGCCCCCGGCCATCTC 480**

**PX255372 CATCCTGGGTGCAATCAATTTTATTACAACCATTATTAATATGAAGCCCCCGGCCATCTC 480**

**PX255373 CATCCTGGGTGCAATCAATTTTATTACAACCATTATTAATATGAAGCCCCCGGCCATCTC 477**

**** ** ** ** ***** ** ***** ** ** ***** ** ** ***** **********

**PX255359 GCAGTATCAAACACCTTTATTCGTATGAGCCACTTTAATTACAGCTGTCCTCCTACTACT 534**

**PX255360 GCAGTATCAAACACCTTTATTCGTATGAGCCACTTTAATTACAGCTGTCCTCCTACTACT 534**

**PX255361 GCAGTATCAAACACCTTTATTCGTATGAGCCACTTTAATTACAGCTGTCCTCCTACTACT 539**

**PX255371 ACAATATCAAACACCCCTGTTTGTATGGGCCATCCTAATTACAGCAGTACTTCTACTCCT 540**

**PX255372 ACAATATCAAACACCCCTGTTTGTATGGGCCATCCTAATTACAGCAGTACTTCTACTCCT 540**

**PX255373 ACAATATCAAACACCCCTGTTTGTATGGGCCATCCTAATTACAGCAGTACTTCTACTCCT 537**

**** *********** * ** ***** **** ********** ** ** ***** ****

**PX255359 ATCCCTTCCAGTCCTGGCCGCCGGTATCACAATATTACTAACAGACCGAAATTTAAACAC 594**

**PX255360 ATCCCTTCCAGTCCTGGCCGCCGGTATCACAATATTACTAACAGACCGAAATTTAAACAC 594**

**PX255361 ATCCCTTCCAGTCCTGGCCGCCGGTATCACAATATTACTAACAGACCGAAATTTAAACAC 599**

**PX255371 ATCTCTACCAGTGCTGGCGGCGGGGATTACAATGTTATTAACCGACCGAAACCTAAACAC 600**

**PX255372 ATCTCTACCAGTGCTGGCGGCGGGGATTACAATGTTATTAACCGACCGAAACCTAAACAC 600**

**PX255373 ATCTCTACCAGTGCTGGCGGCGGGGATTACAATGTTATTAACCGACCGAAACCTAAACAC 597**

***** ** ***** ***** ** ** ** ***** *** **** ******** *********

**PX255359 CACATTCTTCGACCCCGCAGGAGGAGGAGACCCTATCCTATACCAGCAC----------- 643**

**PX255360 CACATTCTTCGACCCCGCAGGAGGAGGAGACCCTATCCTATACCAGCAC----------- 643**

**PX255361 CACATTCTTCGACCCCGCAGGAGGAGGAGACCCTATCCTATACCAGCAC----------- 648**

**PX255371 TACTTTCTTTGACCCCGCAGGAGGAGGAGACCCAATTCTTTATCAACACCTCTTCTGATT 660**

**PX255372 TACTTTCTTTGACCCCGCAGGAGGAGGAGACCCAATTCTTTATCAACACCTCTTCTGATT 660**

**PX255373 TACTTTCTTTGACCCCGCAGGAGGAGGAGACCCAATTCTTTATCAACACCTCTTCTGA-- 655**

**** ***** *********************** ** ** ** ** *****

**PX255359 -------------- 643**

**PX255360 -------------- 643**

**PX255361 -------------- 648**

**PX255371 CTTCGGCCACCAAA 674**

**PX255372 CTTCGGCCACCAAA 674**

**PX255373 -------------- 655**

**Figure S3.** Alignment of sequencing result for PCR product from six fish samples of *Bagrus bajad* from Lake Nasser and the River Nile, generated by Clustal Omega. Identities and similarities are indicated by star (*) and gaps are indicated by dashes (-).

**PX255362 ATCTAGTATTTGGTGCTTGAGCCGGAATAGTAGGAACCGCGCTAAGCCTCCTAATTCGGG 60**

**PX255364 ATCTAGTATTTGGTGCTTGAGCCGGAATAGTAGGAACCGCGCTAAGCCTCCTAATTCGGG 60**

**PX255363 ATCTAGTATTTGGTGCTTGAGCCGGAATAGTAGGAACCGCGCTAAGCCTCCTAATTCGGG 60**

****************************************************************

**PX255362 CAGAACTAAGCCAGCCCGGCTCTCTCCTCGGAGACGACCAGATTTATAATGTAATTGTTA 120**

**PX255364 CAGAACTAAGCCAGCCCGGCTCTCTCCTCGGAGACGACCAGATTTATAATGTAATTGTTA 120**

**PX255363 CAGAACTAAGCCAGCCCGGCTCTCTCCTCGGAGACGACCAGATTTATAATGTAATTGTTA 120**

****************************************************************

**PX255362 CAGCACATGCTTTTGTAATAATTTTCTTTATAGTAATGCCAATTATGATTGGAGGCTTTG 180**

**PX255364 CAGCACATGCTTTTGTAATAATTTTCTTTATAGTAATGCCAATTATGATTGGAGGCTTTG 180**

**PX255363 CAGCACATGCTTTTGTAATAATTTTCTTTATAGTAATGCCAATTATGATTGGAGGCTTTG 180**

****************************************************************

**PX255362 GAAACTGACTAGTACCACTCATGATTGGTGCCCCAGATATGGCCTTCCCTCGAATGAACA 240**

**PX255364 GAAACTGACTAGTACCACTCATGATTGGTGCCCCAGATATGGCCTTCCCTCGAATGAACA 240**

**PX255363 GAAACTGACTAGTACCACTCATGATTGGTGCCCCAGATATGGCCTTCCCTCGAATGAACA 240**

****************************************************************

**PX255362 ACATGAGTTTCTGACTCCTCCCTCCCTCATTCCTCCTCCTCCTCGCCTCATCTGGAGTCG 300**

**PX255364 ACATGAGTTTCTGACTCCTCCCTCCCTCATTCCTCCTCCTCCTCGCCTCATCTGGAGTCG 300**

**PX255363 ACATGAGTTTCTGACTCCTCCCTCCCTCATTCCTCCTCCTCCTCGCCTCATCTGGAGTCG 300**

****************************************************************

**PX255362 AAGCAGGTGCCGGCACAGGGTGAACTGTTTACCCCCCGCTCGCAGGCAATCTTGCCCATG 360**

**PX255364 AAGCAGGTGCCGGCACAGGGTGAACTGTTTACCCCCCGCTCGCAGGCAATCTTGCCCATG 360**

**PX255363 AAGCAGGTGCCGGCACAGGGTGAACTGTTTACCCCCCGCTCGCAGGCAATCTTGCCCATG 360**

****************************************************************

**PX255362 CTGGGCCTTCTGTCGACTTAACCATCTTCTCCCTCCACTTGGCCGGGGTATCATCTATTT 420**

**PX255364 CTGGGCCTTCTGTCGACTTAACCATCTTCTCCCTCCACTTGGCCGGGGTATCATCTATTT 420**

**PX255363 CTGGACCTTCTGTCGACTTAACCATCTTCTCCCTCCACTTGGCCGGGGTATCATCTATTT 420**

****** *********************************************************

**PX255362 TAGGCGCAATTAATTTCATTACAACAATCATTAACATGAAACCCCCCGCCATCTCTCAAT 480**

**PX255364 TAGGCGCAATTAATTTCATTACAACAATCATTAACATGAAACCCCCCGCCATCTCTCAAT 480**

**PX255363 TAGGCGCAATTAATTTCATTACAACAATCATTAACATGAAACCCCCCGCCATCTCTCAAT 480**

****************************************************************

**PX255362 ATCAGACACCCCTATTTGTATGGTCCGTTCTAATTACAGCAGTATTACTTCTTCTATCCC 540**

**PX255364 ATCAGACACCCCTATTTGTATGGTCCGTTCTAATTACAGCAGTATTACTTCTTCTATCCC 540**

**PX255363 ATCAGACACCCCTATTTGTATGGTCCGTTCTAATTACAGCAGTATTACTTCTTCTATCCC 540**

****************************************************************

**PX255362 TACCCGTTCTTGCCGCCGGCATCACAATACTTCTCACAGACCGAAACCTAAACACAACCT 600**

**PX255364 TACCCGTTCTTGCCGCCGGCATCACAATACTTCTCACAGACCGAAACCTAAACACAACCT 600**

**PX255363 TACCCGTTCTTGCCGCCGGCATCACAATACTTCTCACAGACCGAAACCTAAACACAACCT 600**

****************************************************************

**PX255362 TCTTTGATCCTGCCGGAGGAGGAGACCCCATCCTTTACCAACACTTATTCTGATTCTTCG 660**

**PX255364 TCTTTGATCCTGCCGGAGGAGGAGACCCCATCCTTTACCAACACTTATTCTGATTCTTCG 660**

**PX255363 TCTTTGATCCTGCCGGAGGAGGAGACCCCATCCTTTACCAACACTTATTCTGATTCTTCG 660**

****************************************************************

**Figure S4.** Alignment of sequencing result for PCR product from three fish samples of *Sarotherodon galilaeus* from Lake Nasser, generated by Clustal Omega. Identities and similarities are indicated by star (*) and gaps are indicated by dashes (-).

**PX255365 ACTTTATTTAGTATTTGGTGCCTGGGCCGGAATAGTTGGCACAGCCCTCAGCCTACTAAT 60**

**PX255366 -CTTTATTTAGTATTTGGTGCCTGGGCCGGAATAGTTGGCACAGCCCTCAGCCTACTAAT 59**

**PX255367 -CTTTATTTAGTATTTGGTGCCTGGGCCGGAATAGTTGGCACAGCCCTCAGCCTACTAAT 59**

***************************************************************

**PX255365 TCGGGCTGAGTTAGCCCAACCTGGGGCCCTTCTAGGCGATGACCAAATTTACAATGTAAT 120**

**PX255366 TCGGGCTGAGTTAGCCCAACCTGGGGCCCTTCTAGGCGATGACCAAATTTACAATGTAAT 119**

**PX255367 TCGGGCTGAGTTAGCCCAACCTGGGGCCCTTCTAGGCGATGACCAAATTTACAATGTAAT 119**

****************************************************************

**PX255365 TGTCACCGCCCATGCCTTCGTAATAATTTTCTTTATAGTAATACCAATTATGATCGGAGG 180**

**PX255366 TGTCACCGCCCATGCCTTCGTAATAATTTTCTTTATAGTAATACCAATTATGATCGGAGG 179**

**PX255367 TGTCACCGCCCATGCCTTCGTAATAATTTTCTTTATAGTAATACCAATTATGATCGGAGG 179**

****************************************************************

**PX255365 CTTCGGAAACTGACTCGTCCCACTAATAATTGGAGCGCCCGATATAGCATTCCCCCGAAT 240**

**PX255366 CTTCGGAAACTGACTCGTCCCACTAATAATTGGAGCGCCCGATATAGCATTCCCCCGAAT 239**

**PX255367 CTTCGGAAACTGACTCGTCCCACTAATAATTGGAGCGCCCGATATAGCATTCCCCCGAAT 239**

****************************************************************

**PX255365 AAATAACATAAGCTTTTGACTACTGCCGCCCTCCTTCCTACTACTTCTCTCCTCATCCGG 300**

**PX255366 AAATAACATAAGCTTTTGACTATTGCCGCCCTCCTTCCTACTACTTCTCTCCTCATCCGG 299**

**PX255367 AAATAACATAAGCTTTTGACTATTGCCGCCCTCCTTCCTACTACTTCTCTCCTCATCCGG 299**

************************ ***************************************

**PX255365 AGTTGAGGCTGGGGCAGGAACGGGATGAACCGTCTACCCCCCACTTGCCAGCAACATTGC 360**

**PX255366 AGTTGAGGCTGGGGCAGGAACGGGATGAACCGTCTACCCCCCACTTGCCAGCAACATTGC 359**

**PX255367 AGTTGAGGCTGGGGCAGGAACGGGATGAACCGTCTACCCCCCACTTGCCAGCAACATTGC 359**

****************************************************************

**PX255365 ACATGCGGGGGCCTCTGTAGACTTAACTATCTTCTCTCTTCACCTGGCAGGTGTATCATC 420**

**PX255366 ACATGCGGGGGCCTCTGTAGACTTAACTATCTTCTCTCTTCACCTGGCAGGTGTATCATC 419**

**PX255367 ACATGCGGGGGCCTCTGTAGACTTAACTATCTTCTCTCTTCACCTGGCAGGTGTATCATC 419**

****************************************************************

**PX255365 TATTCTAGGAGCAATCAACTTCATCACAACTATTATTAATATAAAACCCCCAGCCATCTC 480**

**PX255366 TATTCTAGGAGCAATCAACTTCATCACAACTATTATTAATATAAAACCCCCAGCCATCTC 479**

**PX255367 TATTCTAGGAGCAATCAACTTCATCACAACTATTATTAATATAAAACCCCCAGCCATCTC 479**

****************************************************************

**PX255365 ACAATACCAGACACCTCTCTTTGTGTGAGCCATCCTAATTACAGCTGTTCTTTTATTACT 540**

**PX255366 ACAATACCAGACACCTCTCTTTGTGTGAGCCATCCTAATTACAGCTGTTCTTTTATTACT 539**

**PX255367 ACAATACCAGACACCTCTCTTTGTGTGAGCCATCCTAATTACAGCTGTTCTTTTATTACT 539**

****************************************************************

**PX255365 ATCCCTCCCAGTACTGGCCGCCGGTATTACAATACTACTAACGGACCGAAACCTAAATAC 600**

**PX255366 ATCCCTCCCAGTACTGGCCGCCGGTATTACAATACTACTAACGGACCGAAACCTAAATAC 599**

**PX255367 ATCCCTCCCAGTACTGGCCGCCGGTATTACAATACTACTAACGGACCGAAACCTAAATAC 599**

****************************************************************

**PX255365 CACCTTCTTTGACCCTGCTGGGGGCGGAGACCCAATCCTTTATCAACACCTT--- 652**

**PX255366 CACCTTCTTTGACCCTGCTGGGGGCGGAGACCCAATCCTTTATCAACACCTTTTC 654**

**PX255367 CACCTTCTTTGACCCTGCTGGGGGCGGAGACCCAATCCTTTATCAACACCTT--- 651**

********************************************************

**Figure S5.** Alignment of sequencing result for PCR product from three fish samples of [*Auchenoglanis occidentalis*](https://www.ncbi.nlm.nih.gov/Taxonomy/Browser/wwwtax.cgi?id=390418) from Lake Nasser, generated by Clustal Omega. Identities and similarities are indicated by star (*) and gaps are indicated by dashes (-).

**PX255368 ---------------------------------CTAAGCCTACTTATCCGAGCAGAACTT 27**

**PX255369 ---------------------------------CTAAGCCTACTTATCCGAGCAGAACTT 27**

**PX255370 TTTGGTGCTTGAGCCGGAATAGTGGGCACAGCCCTAAGCCTACTTATCCGAGCAGAACTT 60**

*******************************

**PX255368 AGCCAACCTGGCGCACTCCTAGGAGACGACCAGATCTATAACGTAATCGTTACCGCCCAC 87**

**PX255369 AGCCAACCTGGCGCACTCCTAGGAGACGACCAGATCTATAACGTAATCGTTACCGCCCAC 87**

**PX255370 AGCCAACCTGGCGCACTCCTAGGAGACGACCAGATCTATAACGTAATCGTTACCGCCCAC 120**

****************************************************************

**PX255368 GCTTTCGTAATAATTTTCTTTATAGTAATACCAATTATGATCGGAGGCTTTGGAAACTGA 147**

**PX255369 GCTTTCGTAATAATTTTCTTTATAGTAATACCAATTATGATCGGAGGCTTTGGAAACTGA 147**

**PX255370 GCTTTCGTAATAATTTTCTTTATAGTAATACCAATTATGATCGGAGGCTTTGGAAACTGA 180**

****************************************************************

**PX255368 CTTATTCCACTAATAATTGGAGCCCCAGACATAGCATTCCCCCGAATAAATAATATGAGT 207**

**PX255369 CTTATTCCACTAATAATTGGAGCCCCAGACATAGCATTCCCCCGAATAAATAATATGAGT 207**

**PX255370 CTTATTCCACTAATAATTGGAGCCCCAGACATAGCATTCCCCCGAATAAATAATATGAGT 240**

****************************************************************

**PX255368 TTCTGACTCCTTCCACCCTCTTTCCTGCTCCTTCTAGCTTCTTCTGGGGTAGAGGCCGGA 267**

**PX255369 TTCTGACTCCTTCCACCCTCTTTCCTGCTCCTTCTAGCTTCTTCTGGGGTAGAGGCCGGA 267**

**PX255370 TTCTGACTCCTTCCACCCTCTTTCCTGCTCCTTCTAGCTTCTTCTGGGGTAGAAGCCGGA 300**

******************************************************* ********

**PX255368 GCTGGAACCGGTTGAACTGTATATCCCCCTCTCGCTGGAAACCTTGCCCATGCTGGAGCA 327**

**PX255369 GCTGGAACCGGTTGAACTGTATATCCCCCTCTCGCTGGAAACCTTGCCCATGCTGGAGCA 327**

**PX255370 GCTGGAACCGGTTGAACTGTATATCCCCCTCTCGCTGGAAACCTTGCCCATGCTGGAGCA 360**

****************************************************************

**PX255368 TCCGTCGAGCTGACCATCTTTTCACTTCACTTAGCAGGAATTTCCTCAATTCTCGGGGCC 387**

**PX255369 TCCGTCGAGCTGACCATCTTTTCACTTCACTTAGCAGGAATTTCCTCAATTCTCGGGGCC 387**

**PX255370 TCCGTCGACCTGACCATCTTTTCACTTCACTTAGCAGGAATTTCCTCAATTCTCGGGGCC 420**

********** *****************************************************

**PX255368 ATCAACTTTATTACTACTATTCTCAATATGAAACCGGCTGCCGTATCTATGTACCAAATT 447**

**PX255369 ATCAACTTTATTACTACTATTCTCAATATGAAACCGGCTGCCGTATCTATGTACCAAATT 447**

**PX255370 ATCAACTTTATTACTACTATTCTCAATATGAAACCGGCTGCCGTATCTATGTACCAAATT 480**

****************************************************************

**PX255368 CCTCTGTTCGTCTGAGCAGTATTAATTACAGCTGTGCTGCTCCTTCTTTCCCTCCCAGTT 507**

**PX255369 CCTCTGTTCGTCTGAGCAGTATTAATTACAGCTGTGCTGCTCCTTCTTTCCCTCCCAGTT 507**

**PX255370 CCTCTGTTCGTCTGAGCAGTATTAATTACAGCTGTGCTGCTCCTTCTTTCCCTCCCAGTT 540**

****************************************************************

**PX255368 TTAGCCGCAGGTATCACAATACTCCTTACAGACCGAAATCTAAATACAGCATTCTTTGAC 567**

**PX255369 TTAGCCGCAGGTATCACAATACTCCTTACAGACCGAAATCTAAATACAGCATTCTTTGAC 567**

**PX255370 TTAGCCGCAGGTATCACAATACTCCTTACAGACCGAAATCTAAATACAGCATTCTTTGAC 600**

****************************************************************

**PX255368 CCCGCCGGAGGAGGAGACCCTATTCTCTATCAACACCTATTCTGATTCTTCGGCCAC--- 624**

**PX255369 CCCGCCGGAGGAGGAGACCCTATTCTCTATCAACACCTATTCTGATTCTTCGGCCAC--- 624**

**PX255370 CCCGCCGGAGGAGGAGACCCTATTCTCTATCAACACCTATTCTGATTCTTCGGCCACCCA 660**

*************************************************************

**Figure S6.** Alignment of sequencing result for PCR product from three fish samples of *Lates niloticus* from Lake Nasser, generated by Clustal Omega. Identities and similarities are indicated by star (*) and gaps are indicated by dashes (-).

**PX255374 CCTTTATCTTGTATTTGGTGCTTGAGCCGGAATGGTTGGGACTGCCCTTAGCCTATTAAT 60**

**PX255375 -CTTTATCTTGTATTTGGTGCTTGAGCCGGAATGGTTGGGACTGCCCTTAGCCTATTAAT 59**

**PX255376 -CTTTATCTTGTATTTGGTGCTTGAGCCGGAATGGTTGGGACTGCCCTTAGCCTATTAAT 59**

***************************************************************

**PX255374 TCGAGCAGAGCTAAGTCAGCCCGGATCTCTCCTCGGGGACGACCAGATTTACAATGTTAT 120**

**PX255375 TCGAGCAGAGCTAAGTCAGCCCGGATCTCTCCTCGGGGACGACCAGATTTACAATGTTAT 119**

**PX255376 TCGAGCAGAGCTAAGTCAGCCCGGATCTCTCCTCGGGGACGACCAGATTTACAATGTTAT 119**

****************************************************************

**PX255374 CGTTACAGCACATGCATTTGTAATAATCTTTTTCATGGTCATACCAATTATAATTGGCGG 180**

**PX255375 CGTTACAGCACATGCATTTGTAATAATCTTTTTCATGGTCATACCAATTATAATTGGCGG 179**

**PX255376 CGTTACAGCACATGCATTTGTAATAATCTTTTTCATGGTCATACCAATTATAATTGGCGG 179**

****************************************************************

**PX255374 CTTTGGGAATTGACTCGTACCCCTGATAATTGGTGCCCCCGATATAGCATTTCCACGAAT 240**

**PX255375 CTTTGGGAATTGACTCGTACCCCTGATAATTGGTGCCCCCGATATAGCATTTCCACGAAT 239**

**PX255376 CTTTGGGAATTGACTCGTACCCCTGATAATTGGTGCCCCCGATATAGCATTTCCACGAAT 239**

****************************************************************

**PX255374 AAATAATATGAGCTTCTGACTTCTTCCTCCATCTTTCCTTCTCCTCTTAGCCTCTTCAGG 300**

**PX255375 AAATAATATGAGCTTCTGACTTCTTCCTCCATCTTTCCTTCTCCTCTTAGCCTCTTCAGG 299**

**PX255376 AAATAATATGAGCTTCTGACTTCTTCCTCCATCTTTCCTTCTCCTCTTAGCCTCTTCAGG 299**

****************************************************************

**PX255374 CGTGGAAGCAGGGGCCGGAACAGGCTGAACAGTTTATCCTCCCCTCGCTGGAAATCTTGC 360**

**PX255375 CGTGGAAGCAGGGGCCGGAACAGGCTGAACAGTTTATCCTCCCCTCGCTGGAAATCTTGC 359**

**PX255376 CGTGGAAGCAGGGGCCGGAACAGGCTGAACAGTTTATCCTCCCCTCGCTGGAAATCTTGC 359**

****************************************************************

**PX255374 CCACGCAGGAGCATCCGTTGATTTAACTATTTTTTCACTACATCTTGCAGGTGTCTCTTC 420**

**PX255375 CCACGCAGGAGCATCCGTTGATTTAACTATTTTTTCACTACATCTTGCAGGTGTCTCTTC 419**

**PX255376 CCACGCAGGAGCATCCGTTGATTTAACTATTTTTTCACTACATCTTGCAGGTGTCTCTTC 419**

****************************************************************

**PX255374 CATTCTTGGTGCAATTAACTTCATTACTACTATTGTAAACATAAAACCCCCTGCCATCTC 480**

**PX255375 CATTCTTGGTGCAATTAACTTCATTACTACTATTGTAAACATAAAACCCCCTGCCATCTC 479**

**PX255376 CATTCTTGGTGCAATTAACTTCATTACTACTATTGTAAACATAAAACCCCCTGCCATCTC 479**

****************************************************************

**PX255374 ACAGTACCAAACCCCCTTATTTGTTTGGGCTGTTTTAATTACAGCTGTCCTATTACTACT 540**

**PX255375 ACAGTACCAAACCCCCTTATTTGTTTGGGCTGTTTTAATTACAGCTGTCCTATTACTACT 539**

**PX255376 ACAGTACCAAACCCCCTTATTTGTTTGGGCTGTTTTAATTACAGCTGTCCTATTACTACT 539**

****************************************************************

**PX255374 ATCCCTTCCCGTTCTGGCCGCAGGCATTACTATACTACTAACGGACCGAAATTTAAACAC 600**

**PX255375 ATCCCTTCCCGTTCTGGCCGCAGGCATTACTATACTACTAACGGACCGAAATTTAAACAC 599**

**PX255376 ATCCCTTCCCGTTCTGGCCGCAGGCATTACTATACTACTAACGGACCGAAATTTAAACAC 599**

****************************************************************

**PX255374 TACCTTTTTTGACCCTGCCGGAGGAGGAGACCCAATTCTTTATCAACACTTG 652**

**PX255375 TACCTTTTTTGACCCTGCCGGAGGAGGAGACCCAATTCTTTATCAACACTTG 651**

**PX255376 TACCTTTTTTGACCCTGCCGGAGGAGGAGACCCAATTCTTTATCAACACTTG 651**

********************************************************

**Figure S7.** Alignment of sequencing result for PCR product from three fish samples of *Sardinella tawilis* from Lake Nasser, generated by Clustal Omega. Identities and similarities are indicated by star (*) and gaps are indicated by dashes (-).

**PX255377 TCAACCAACCACAAAGACATTGGCACCCTCTATCTAGTATTTGGTGCTTGGGCCGGAATA 60**

**PX255378 TCAACCAACCACAAAGACATTGGCACCCTCTATCTAGTATTTGGTGCTTGGGCCGGAATA 60**

**PX255379 TCAACCAACCACAAAGACATTGGCACCCTCTATCTAGTATTTGGTGCTTGGGCCGGAATA 60**

****************************************************************

**PX255377 GTAGGAACCGCACTCAGCCTCCTGATCCGAGCAGAACTAAGCCAGCCCGGCTCCCTCCTC 120**

**PX255378 GTAGGAACCGCACTCAGCCTCCTGATCCGAGCAGAACTAAGCCAGCCCGGCTCCCTCCTC 120**

**PX255379 GTAGGAACCGCACTCAGCCTCCTGATCCGAGCAGAACTAAGCCAGCCCGGCTCCCTCCTC 120**

****************************************************************

**PX255377 GGGGACGACCGTATTTATAATGTAATTGTTACGGCACATGCCTTTGTAATAATCTTCTTT 180**

**PX255378 GGGGACGACCGTATTTATAATGTAATTGTTACGGCACATGCCTTTGTAATAATCTTCTTT 180**

**PX255379 GGGGACGACCAGATTTATAATGTAATTGTTACAGCACATGCCTTTGTAATAATTTTCTTT 180**

************ ******************** ******************** ********

**PX255377 ATAATAGTGCCAATTATGATTGGAGGCTTTGGGAACTGATTAGTCCCGCTTATGATCGGC 240**

**PX255378 ATAATAGTGCCAATTATGATTGGAGGCTTTGGGAACTGATTAGTCCCGCTTATGATCGGC 240**

**PX255379 ATAGTAATGCCAATTATGATTGGAGGCTTTGGGAACTGATTAGTCCCACTTATGATCGGC 240**

***** ** **************************************** **************

**PX255377 GCACCAGACATGGCCTTCCCTCGAATAAACAACATAAGCTTCTGACTTCTCCCCCCATCA 300**

**PX255378 GCACCAGACATGGCCTTCCCTCGAATAAACAACATAAGCTTCTGACTTCTCCCCCCATCA 300**

**PX255379 GCACCAGACATGGCCTTCCCTCGAATAAACAACATAAGCTTCTGACTTCTCCCCCCATCA 300**

****************************************************************

**PX255377 TTCCTCCTTCTCCTCGCTTCATCACGGGTCGAAGCGGGCGCCGGTACAGGGTGAACTGTC 360**

**PX255378 TTCCTCCTTCTCCTCGCTTCATCACGGGTCGAAGCGGGCGCCGGTACAGGGTGAACTGTC 360**

**PX255379 TTCCTCCTTCTCCTCGCTTCATCAGGGGTCGAAGCGGGCGCCGGTACAGGGTGAACTGTC 360**

************************** *************************************

**PX255377 TACCCCCCACTCGCAGGCAATCTCGCCCACGCTGGGCCCTCTGTTGACCTAACCATCTTC 420**

**PX255378 TACCCCCCACTCGCAGGCAATCTCGCCCACGCTGGGCCCTCTGTTGACCTAACCATCTTC 420**

**PX255379 TACCCCCCACTCGCAGGCAATCTCGCCCACGCTGGGCCCTCTGTTGACCTAACCATCTTC 420**

****************************************************************

**PX255377 TCACTCCACCTAGCCGGGGTATCATCTATTCTAGGGGCAATTAATTTCATTACAACAATT 480**

**PX255378 TCACTCCACCTAGCCGGGGTATCATCTATTCTAGGGGCAATTAATTTCATTACAACAATT 480**

**PX255379 TCACTCCACCTAGCCGGGGTATCATCTATTCTAGGGGCAATTAATTTCATTACAACAATT 480**

****************************************************************

**PX255377 ATTAACATGAAGCCCCCTGCTATCTCTCAGTACCAAACGCCCCTGTTCGTGTGGTCCGTC 540**

**PX255378 ATTAACATGAAGCCCCCTGCTATCTCTCAGTACCAAACGCCCCTGTTCGTGTGGTCCGTC 540**

**PX255379 ATTAACATGAAGCCCCCTGCTATCTCTCAGTACCAAACGCCCCTGTTCGTGTGGTCCGTC 540**

****************************************************************

**PX255377 CTAATTACGGCGGTGCTGCTTCTTCTGTCACTGCCTGTTCTTGCTGCCGGCATTACAATG 600**

**PX255378 CTAATTACGGCGGTGCTGCTTCTTCTGTCACTGCCTGTTCTTGCTGCCGGCATTACAATG 600**

**PX255379 CTAATTACGGCGGTGCTGCTTCTTCTGTCACTGCCTGTTCTTGCTGCCGGCATTACAATG 600**

****************************************************************

**PX255377 CTTCTCACAGACCGAAATCTAAACACGACCTTCTTTGATCCTGCCGGGGGAGGGGATCCC 660**

**PX255378 CTTCTCACAGACCGAAATCTAAACACGACCTTCTTTGATCCTGCCGGGGGAGGGGATCCC 660**

**PX255379 CTTCTCACAGACCGAAATCTAAACACGACCTTCTTTGATCCTGCCGGGGGAGGGGATCCC 660**

****************************************************************

**PX255377 ATCCTCTACCAACATTTATTCTGATTCTTCGGTCACCCTGAAGT 704**

**PX255378 ATCCTCTACCAACATTTATTCTGATTCTTCGGTCACCCTGAAGT 704**

**PX255379 ATCCTCTACCAACATTTATTCTGATTCTT--------------- 689**

*********************************

**Figure S8.** Alignment of sequencing result for PCR product from three fish samples of *Coptodon zillii* from Lake Nasser, generated by Clustal Omega. Identities and similarities are indicated by star (*) and gaps are indicated by dashes (-).

**PX255374 --------------------------CCTTTATCTTGTATTTGGTGCTTGAGCCGGAATG 34**

**PX255373* --------------------------CCTTTACATAGCAATTGGTGCTTGAGCCGGCATA 34**

**PX255361* ---------------------------CTCTACCTAGTATTTGGTGCCTGAGCCGGAATA 33**

**PX255366 ---------------------------CTTTATTTAGTATTTGGTGCCTGGGCCGGAATA 33**

**PX255368 ------------------------------------------------------------ 0**

**PX255379 TCAACCAACCACAAAGACATTGGCACCCTCTATCTAGTATTTGGTGCTTGGGCCGGAATA 60**

**PX255355 TCAACCAACCACAAGGATATGGGCACCCTCTATCTAGTATTTGGTGCTTGAGCCGGAATA 60**

**PX255357* TCAACCAACCACAAGGATATTGGCACCCTCTATCTAGTATTTGGTGCTTGAGCCGGAATA 60**

**PX255382* TCAACCAACCACAAGGATATGGGCACCCTCTATCTAGTATTTGGTGCTTGAGCCGGAATA 60**

**PX255363 -------------------------------ATCTAGTATTTGGTGCTTGAGCCGGAATA 29**

**PX255374 GTTGGGACTGCCCTTAGCCTATTAATTCGAGCAGAGCTAAGTCAGCCCGGATCTCTCCTC 94**

**PX255373* GTGG---GTACGGTCAGCCTATTAATTCGGGCAGAACTAGCCCAACCCGGAGCCCTTCTG 91**

**PX255361* GTCGGCACAGCCCTAAGCCTACTTATTCGGGCAGAGCTAGCCCAACCTGGCGCTCTCCTG 93**

**PX255366 GTTGGCACAGCCCTCAGCCTACTAATTCGGGCTGAGTTAGCCCAACCTGGGGCCCTTCTA 93**

**PX255368 ------------CTAAGCCTACTTATCCGAGCAGAACTTAGCCAACCTGGCGCACTCCTA 48**

**PX255379 GTAGGAACCGCACTCAGCCTCCTGATCCGAGCAGAACTAAGCCAGCCCGGCTCCCTCCTC 120**

**PX255355 GTAGGAACTGCACTAAGCCTCCTAATTCGGGCAGAACTAAGCCAGCCCGGCTCTCTTCTC 120**

**PX255357* GTAGGAACTGCACTAAGCCTCCTAATTCGGGCAGAACTAAGCCAGCCCGGCTCTCTTCTC 120**

**PX255382* GTAGGAACTGCACTAAGCCTCCTAATTCGGGCAGAACTAAGCCAGCCCGGCTCTCTTCTC 120**

**PX255363 GTAGGAACCGCGCTAAGCCTCCTAATTCGGGCAGAACTAAGCCAGCCCGGCTCTCTCCTC 89**

*** ***** * ** ** ** ** * ** ** ** * ** ****

**PX255374 GGGGACGACCAGATTTACAATGTTATCGTTACAGCACATGCATTTGTAATAATCTTTTTC 154**

**PX255373* GGCGACGACCAGATTTATAATGTAATTGTTACTGCTCATGCCTTTGTAATAATTTTCTTT 151**

**PX255361* GGAGACGACCAAATTTATAATGTTATTGTTACTGCCCATGCCTTCGTAATAATCTTCTTT 153**

**PX255366 GGCGATGACCAAATTTACAATGTAATTGTCACCGCCCATGCCTTCGTAATAATTTTCTTT 153**

**PX255368 GGAGACGACCAGATCTATAACGTAATCGTTACCGCCCACGCTTTCGTAATAATTTTCTTT 108**

**PX255379 GGGGACGACCAGATTTATAATGTAATTGTTACAGCACATGCCTTTGTAATAATTTTCTTT 180**

**PX255355 GGAGACGACCAAATCTATAATGTAATTGTTACAGCACATGCTTTCGTAATAATTTTCTTT 180**

**PX255357* GGAGACGACCAAATCTATAATGTAATTGTTACAGCACATGCTTTCGTAATAATTTTCTTT 180**

**PX255382* GGAGACGACCAAATCTATAATGTAATTGTTACAGCACATGCTTTCGTAATAATTTTCTTT 180**

**PX255363 GGAGACGACCAGATTTATAATGTAATTGTTACAGCACATGCTTTTGTAATAATTTTCTTT 149**

**** ** ***** ** ** ** ** ** ** ** ** ** ** ** ******** ** ****

**PX255374 ATGGTCATACCAATTATAATTGGCGGCTTTGGGAATTGACTCGTACCCCTGATAATTGGT 214**

**PX255373* ATAGTAATGCCAATCATAATTGGAGGCTTTGGGAACTGGCTGGTTCCACTAATGATTGGA 211**

**PX255361* ATAGTAATACCAATTATGATTGGAGGCTTTGGAAACTGACTTATCCCACTAATAATCGGA 213**

**PX255366 ATAGTAATACCAATTATGATCGGAGGCTTCGGAAACTGACTCGTCCCACTAATAATTGGA 213**

**PX255368 ATAGTAATACCAATTATGATCGGAGGCTTTGGAAACTGACTTATTCCACTAATAATTGGA 168**

**PX255379 ATAGTAATGCCAATTATGATTGGAGGCTTTGGGAACTGATTAGTCCCACTTATGATCGGC 240**

**PX255355 ATAGTAATACCAATTATGATTGCATGCTTTGGAAGTTGACAAGTTCCTTTTATGATTGGT 240**

**PX255357* ATAGTAATACCAATTATGATTGGAGGCTTTGGAAACTGACTAGTACCCCTCATGATTGGT 240**

**PX255382* ATAGTAATACCAATTATGATTGGAGGCTTTGGAAACTGACTAGTACCCCTCATGATTGGT 240**

**PX255363 ATAGTAATGCCAATTATGATTGGAGGCTTTGGAAACTGACTAGTACCACTCATGATTGGT 209**

**** ** ** ***** ** ** * **** ** * ** * ** * ** ** ****

**PX255374 GCCCCCGATATAGCATTTCCACGAATAAATAATATGAGCTTCTGACTTCTTCCTCCATCT 274**

**PX255373* GCACCAGACATGGCCTTTCCCCGAATAAATAACATAAGCTTCTGACTTCTCCCCCCCTCT 271**

**PX255361* GCCCCAGACATAGCATTCCCCCGAATAAATAACATGAGCTTCTGACTACTCCCTCCCTCC 273**

**PX255366 GCGCCCGATATAGCATTCCCCCGAATAAATAACATAAGCTTTTGACTATTGCCGCCCTCC 273**

**PX255368 GCCCCAGACATAGCATTCCCCCGAATAAATAATATGAGTTTCTGACTCCTTCCACCCTCT 228**

**PX255379 GCACCAGACATGGCCTTCCCTCGAATAAACAACATAAGCTTCTGACTTCTCCCCCCATCA 300**

**PX255355 GCAGTTGATGAGGACTTTCCTCGCATAA---GCAAGAACTTTTGACTTCTCCCCCCCTCG 297**

**PX255357* GCACCAGACATGGCCTTCCCTCGAATAAATAACATGAGCTTTTGACTTCTCCCCCCCTCA 300**

**PX255382* GCACCAGACATGGCCTTCCCTCGAATAAATAACATGAGCTTTTGACTTCTCCCCCCCTCA 300**

**PX255363 GCCCCAGATATGGCCTTCCCTCGAATGAACAACATGAGTTTCTGACTCCTCCCTCCCTCA 269**

**** ** * ** ** ** ** * * * ** ***** * ** ** ****

**PX255374 TTCCTTCTCCTCTTAGCCTCTTCAGGCGTGGAAGCAGGGGCCGGAACAGGCTGAACAGTT 334**

**PX255373* TTCTTATTACTGCTTGCCTCATCAGGAGTTGAAGCAGGGGCAGGAACAGGATGAACTGTT 331**

**PX255361* TTCTTACTACTTCTAGCCTCCTCCGGAGTTGAGGCAGGAGCAGGAACAGGATGAACTGTA 333**

**PX255366 TTCCTACTACTTCTCTCCTCATCCGGAGTTGAGGCTGGGGCAGGAACGGGATGAACCGTC 333**

**PX255368 TTCCTGCTCCTTCTAGCTTCTTCTGGGGTAGAGGCCGGAGCTGGAACCGGTTGAACTGTA 288**

**PX255379 TTCCTCCTTCTCCTCGCTTCATCAGGGGTCGAAGCGGGCGCCGGTACAGGGTGAACTGTC 360**

**PX255355 TTTCTTCATCTTCTCTCCTCTTCTTGAGTCGAAGCATGTGCCGGCACATGATGGACTGTT 357**

**PX255357* TTTCTTCTTCTTCTCGCCTCATCTGGAGTCGAAGCAGGTGCCGGCACAGGATGGACTGTT 360**

**PX255382* TTTCTTCTTCTTCTCGCCTCATCTGGAGTCGAAGCAGGTGCCGGCACAGGATGGACTGTT 360**

**PX255363 TTCCTCCTCCTCCTCGCCTCATCTGGAGTCGAAGCAGGTGCCGGCACAGGGTGAACTGTT 329**

**** * ** * * ** ** * ** ** ** * ** ** ** * ** ** ****

**PX255374 TATCCTCCCCTCGCTGGAAATCTTGCCCACGCAGGAGCATCCGTTGATTTAACTATTTTT 394**

**PX255373* TACCCGCCTCTCGCCGGAAACCTTGCTCATGCGGGGGCCTCTGTAGACTTAACCATCTTT 391**

**PX255361* TATCCCCCCCTCGCTGGAAACCTTGCACACGCCGGAGCCTCTGTAGATCTAACTATTTTC 393**

**PX255366 TACCCCCCACTTGCCAGCAACATTGCACATGCGGGGGCCTCTGTAGACTTAACTATCTTC 393**

**PX255368 TATCCCCCTCTCGCTGGAAACCTTGCCCATGCTGGAGCATCCGTCGAGCTGACCATCTTT 348**

**PX255379 TACCCCCCACTCGCAGGCAATCTCGCCCACGCTGGGCCCTCTGTTGACCTAACCATCTTC 420**

**PX255355 TATCCCCCGCTCCCGGGCAATCTTGCCCACGCTGGACCTTCTGTTGACTTAACCATCTTC 417**

**PX255357* TATCCCCCGCTCGCAGGCAATCTTGCCCACGCTGGACCTTCTGTTGACTTAACCATCTTC 420**

**PX255382* TATCCCCCGCTCGCAGGCAATCTTGCCCACGCTGGACCTTCTGTTGACTTAACCATCTTC 420**

**PX255363 TACCCCCCGCTCGCAGGCAATCTTGCCCATGCTGGACCTTCTGTCGACTTAACCATCTTC 389**

**** ** ** ** * * ** * ** ** ** ** * ** ** ** * ** ** ****

**PX255374 TCACTACATCTTGCAGGTGTCTCTTCCATTCTTGGTGCAATTAACTTCATTACTACTATT 454**

**PX255373* TCACTCCATCTTGCAGGTGTTTCCTCCATCCTGGGTGCAATCAATTTTATTACAACCATT 451**

**PX255361* TCCCTCCACCTGGCAGGTGTATCTTCTATTCTAGGGGCCATCAACTTCATTACCACAATC 453**

**PX255366 TCTCTTCACCTGGCAGGTGTATCATCTATTCTAGGAGCAATCAACTTCATCACAACTATT 453**

**PX255368 TCACTTCACTTAGCAGGAATTTCCTCAATTCTCGGGGCCATCAACTTTATTACTACTATT 408**

**PX255379 TCACTCCACCTAGCCGGGGTATCATCTATTCTAGGGGCAATTAATTTCATTACAACAATT 480**

**PX255355 TCCCTCAACTTGGCCGGAATGTTATCTATTTTATGTGCTTTTAATTTTATAACAACCATT 477**

**PX255357* TCCCTCCACTTGGCCGGAGTGTCATCTATTTTAGGTGCAATTAATTTTATCACAACCATT 480**

**PX255382* TCCCTCCACTTGGCCGGAGTGTCATCTATTTTAGGTGCAATTAATTTTATCACAACCATT 480**

**PX255363 TCCCTCCACTTGGCCGGGGTATCATCTATTTTAGGCGCAATTAATTTCATTACAACAATC 449**

**** ** * * ** ** * * ** ** * * ** * ** ** ** ** ** ****

**PX255374 GTAAACATAAAACCCCCTGCCATCTCACAGTACCAAACCCCCTTATTTGTTTGGGCTGTT 514**

**PX255373* ATTAATATGAAGCCCCCGGCCATCTCACAATATCAAACACCCCTGTTTGTATGGGCCATC 511**

**PX255361* ATTAACATAAAACCCCCAGCCATCTCGCAGTATCAAACACCTTTATTCGTATGAGCCACT 513**

**PX255366 ATTAATATAAAACCCCCAGCCATCTCACAATACCAGACACCTCTCTTTGTGTGAGCCATC 513**

**PX255368 CTCAATATGAAACCGGCTGCCGTATCTATGTACCAAATTCCTCTGTTCGTCTGAGCAGTA 468**

**PX255379 ATTAACATGAAGCCCCCTGCTATCTCTCAGTACCAAACGCCCCTGTTCGTGTGGTCCGTC 540**

**PX255355 ATTAACATGAAACCCCCTGCCGTCTCCCAATATCAAACACCCCTATTTGTGTGATCCGTC 537**

**PX255357 ATTAACATGAAACCCCCTGCCATCTCCCAATATCAAACACCCCTATTTGTGTGATCCGTC 540**

**PX255382* ATTAACATGAAACCCCCTGCCATCTCCCAATATCAAACACCCCTATTTGTGTGATCCGTC 540**

**PX255363* ATTAACATGAAACCCCCCGCCATCTCTCAATATCAGACACCCCTATTTGTATGGTCCGTT 509**

*** ** ** ** ** * ** * ** ** ** * ** * ** ** ** ***

**PX255374 TTAATTACAGCTGTCCTATTACTACTATCCCTTCCCGTTCTGGCCGCAGGCATTACTATA 574**

**PX255373* CTAATTACAGCAGTACTTCTACTCCTATCTCTACCAGTGCTGGCGGCGGGGATTACAATG 571**

**PX255361* TTAATTACAGCTGTCCTCCTACTACTATCCCTTCCAGTCCTGGCCGCCGGTATCACAATA 573**

**PX255366 CTAATTACAGCTGTTCTTTTATTACTATCCCTCCCAGTACTGGCCGCCGGTATTACAATA 573**

**PX255368 TTAATTACAGCTGTGCTGCTCCTTCTTTCCCTCCCAGTTTTAGCCGCAGGTATCACAATA 528**

**PX255379 CTAATTACGGCGGTGCTGCTTCTTCTGTCACTGCCTGTTCTTGCTGCCGGCATTACAATG 600**

**PX255355 CTAATTACCGCACTACTACTCCTTCTATCCCTGCCCGTTCTTGCCGCCGGCATCACAATA 597**

**PX255357* CTAATTACCGCAGTACTACTCCTTCTATCCCTGCCCGTTCTTGCCGCCGGCATCACAATA 600**

**PX255382* CTAATTACCGCAGTACTACTCCTTCTATCCCTGCCCGTTCTTGCCGCCGGCATCACAATA 600**

**PX255363 CTAATTACAGCAGTATTACTTCTTCTATCCCTACCCGTTCTTGCCGCCGGCATCACAATA 569**

********* ** * * * * ** ** ** ** ** * ** ** ** ** ** ****

**PX255374 CTACTAACGGACCGAAATTTAAACACTACCTTTTTTGACCCTGCCGGAGGAGGAGACCCA 634**

**PX255373* TTATTAACCGACCGAAACCTAAACACTACTTTCTTTGACCCCGCAGGAGGAGGAGACCCA 631**

**PX255361* TTACTAACAGACCGAAATTTAAACACCACATTCTTCGACCCCGCAGGAGGAGGAGACCCT 633**

**PX255366 CTACTAACGGACCGAAACCTAAATACCACCTTCTTTGACCCTGCTGGGGGCGGAGACCCA 633**

**PX255368 CTCCTTACAGACCGAAATCTAAATACAGCATTCTTTGACCCCGCCGGAGGAGGAGACCCT 588**

**PX255379 CTTCTCACAGACCGAAATCTAAACACGACCTTCTTTGATCCTGCCGGGGGAGGGGATCCC 660**

**PX255355 CTTCTAACAGACCGAAACCTTAACACTACATTCTTTGACCCGGCGGGAGGAGGAGACCCA 657**

**PX255357* CTTCTAACAGACCGAAACCTAAACACAACCTTCTTTGACCCTGCCGGAGGAGGAGACCCC 660**

**PX255382* CTTCTAACAGACCGAAACCTAAACACAACCTTCTTTGACCCTGCCGGAGGAGGAGACCCC 660**

**PX255363 CTTCTCACAGACCGAAACCTAAACACAACCTTCTTTGATCCTGCCGGAGGAGGAGACCCC 629**

*** * ** ******** * ** ** * ** ** ** ** ** ** ** ** ** ****

**PX255374 ATTCTTTATCAACACTTG------------------------------ 652**

**PX255373 ATTCTTTATCAACACCTCTTCTGA------------------------ 655**

**PX255361 ATCCTATACCAGCAC--------------------------------- 648**

**PX255366 ATCCTTTATCAACACCTTTTC--------------------------- 654**

**PX255368 ATTCTCTATCAACACCTATTCTGATTCTTCGGCCAC------------ 624**

**PX255379 ATCCTCTACCAACATTTATTCTGATTCTT------------------- 689**

**PX255355 ATTCTTTATCAACACTTATTCTGATTCTTCGGTCACCCTGAAGTGTCA 705**

**PX255357* ATCCTATACCAACACTTATTCTGATTCTTCGGCCACCCAGAAGTT--- 705**

**PX255382* ATCCTATACCAACACTTATTCTGATTCTTCGGCCACCCAGAAGTT--- 705**

**PX255363 ATCCTTTACCAACACTTATTCTGATTCTTCG----------------- 660**

**** ** ** ** ****

**Figure S9.** Alignment of sequencing result for PCR product from ten representative freshwater fish samples from Lake Nasser and the River Nile, generated by Clustal Omega. Identities and similarities are indicated by star (*) and gaps are indicated by dashes (-). ***** The asterisk used due to same fish name.
